# Microbial community characteristics and pathogens detection in *Rhipicephalus sanguineus* and *Haemaphysalis hystricis* from Hainan Island, China

**DOI:** 10.3389/fmicb.2024.1450219

**Published:** 2024-10-08

**Authors:** Chang Shu, Jitrawadee Intirach, Yunfei Zhou, Suzhen Gao, Xin Lv, Huisheng Jiao, Yue Hu, Zhiyue Lv

**Affiliations:** ^1^Hainan Affiliated Hospital of Hainan Medical University, Hainan General Hospital, Haikou, China; ^2^School of Basic Medical Sciences and Life Sciences, Hainan Medical University, Haikou, China; ^3^NHC Key Laboratory of Tropical Disease Control, Hainan Medical University, Haikou, China; ^4^International School of Public Health and One Health, Hainan Medical University, Haikou, China; ^5^Key Laboratory of Tropical Disease Control, Ministry of Education, Sun Yat-sen University, Guangzhou, China

**Keywords:** *Rhipicephalus sanguineus*, *Haemaphysalis hystricis*, microbial community, 16S rRNA, biomarker, tick-borne pathogen, Hainan Island

## Abstract

**Background:**

Microbial communities significantly influence the vector capacity of ticks, which, along with tick-borne diseases, pose an increasing global threat. Due to the substantial individual variability caused by various factors, it is essential to assess tick microbial communities and vectorial capacities under different environmental conditions. However, there is a relative scarcity of research on the microbial communities and pathogen transmission of ticks in different physiological states and environmental conditions, especially in Hainan Island, southern China.

**Methods:**

From 2021 to 2022, we collected 4,167 tick samples, grouping them by blood meal status, developmental stage, sex, time, geographical location, and tick species. We selected 128 samples for full-length 16S rRNA sequencing to describe microbial community characteristics and identify potential biomarkers. Seven hundred seventy-two samples were tested for seven tick-borne pathogens (*Rickettsia*, *Borrelia burgdorferi*, *Ehrlichia*, *Anaplasma*, *Theileria*, *Babesia*, and *Hepatozoon*), and sera from 208 residents of Hainan Island were tested for IgG antibodies against *Rickettsia* and *B. burgdorferi*.

**Results:**

Blood meal status, developmental stage, sex, time, geographical location, and tick species significantly influenced the microbial communities of ticks. We observed distinct microbial community characteristics across different states. We noted the non-random replacement of stable and transient species, with functional differences between parasitic and engorged ticks mainly driven by transient species. Functionally, we observed three distinct response patterns: driven by stable species, transient species, and both together in response to the six factors. We identified 273 potential biomarkers (200 robust core species and 73 robust differential species). Six genera and eight species of pathogens were detected in ticks, with an overall positivity rate of 12.44% (96/772). Among humans, 18.27% (38/208) of serum samples were positive for at least one tick-borne pathogen IgG.

**Conclusion:**

Our findings indicate that these six factors significantly influence both tick microbial communities and vectorial capacity, with varying effects on vector competence for different pathogens and inconsistent impacts on microbial communities under different conditions. This study supplemented the understanding of tick microbial communities on Hainan Island, assessed the relatively high risk of tick-borne pathogens in the region, and evaluated the impact of these factors on both microbial communities and vectorial capacity.

## Graphical abstract

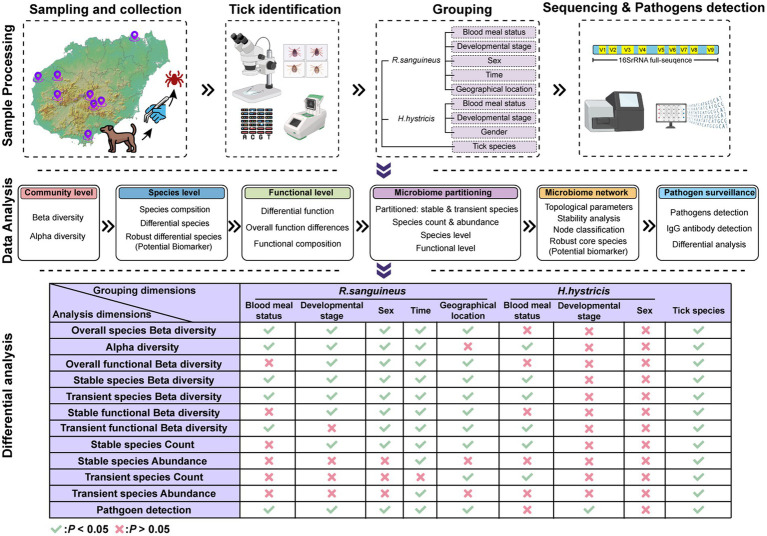

## Introductions

Ticks are recognized as vectors of medically important pathogens due to their extensive host range, widespread distribution, and ability to transmit a variety of pathogens (Klompen et al., 1996; Brites-Neto et al., 2015; Madison-Antenucci et al., 2020; Rochlin and Toledo, 2020). The threat of ticks and tick-borne diseases is increasing, as seen in rising clinical cases, economic impacts, and expanding geographic distribution (Md et al., 2019; Singh et al., 2022; Eisen and Eisen, 2023). While previous studies focus on pathogen positivity rates, microbial community studies provide early insights into tick vectorial competence by influencing pathogen carriage. Research studies have demonstrated that the microbial communities within ticks are closely related to their capacity to carry pathogens, and that these communities vary under different conditions (Chicana et al., 2019; Wu-Chuang et al., 2021). For example, Narasimhan et al. (2014) successfully reduced the colonization rates of *Borrelia burgdorferi* in *Ixodes scapularis* by disrupting its microbial community with antibiotics, finding that ticks with higher microbial diversity had a negatively correlated colonization success rate with *B. burgdorferi*. These findings emphasize the importance of studying tick microbial communities to understand their capability as pathogen vectors (Kim, 2022; Du et al., 2023).

With advancements in sequencing technology, we can now describe the microbial communities of ticks in greater detail. Factors like blood meals, developmental stages, and environmental conditions influence tick microbial communities (Swei and Kwan, 2017; Chicana et al., 2019; Thapa et al., 2019; Brinkerhoff et al., 2020; Gil et al., 2020; Lejal et al., 2021; Wu-Chuang et al., 2021). These studies enhance our understanding of tick microbial communities under varied conditions but also highlight inconsistencies in conclusions due to differences in microbial diversity, tick species, environmental conditions, and methodologies (Zolnik et al., 2016; Swei and Kwan, 2017; Chicana et al., 2019; Kueneman et al., 2021). Such variability contributes to result irreproducibility and complicates cross-study comparisons. Developing effective control strategies requires accumulating microbial community data under various conditions and exploring multiple factors to reduce inconsistencies arising from different research backgrounds. Additionally, conducting nucleic acid or antibody level detection in ticks and humans under as similar research conditions as possible not only reflects the epidemiological status of tick-borne pathogens in the region but also provides data on the actual pathogen positivity rates associated with different microbial community characteristics. This approach helps link microbial community characteristics with true pathogen positivity rates, thereby identifying potential influencing factors in pathogen transmission. Unresolved questions persist, particularly regarding the microbiome of *Rhipicephalus sanguineus*, the most widespread tick species, especially within China, and the almost entirely unexplored microbiome of *Haemaphysalis hystricis*. Current research often focuses on isolated factors affecting microbial communities, lacking a comprehensive evaluation of their interplay and individual contributions. Additionally, as far as we know, the role of environmental bacteria within tick microbiomes has not been described. Furthermore, the identification of biomarkers (specific microorganisms within the tick microbiota that can be used to monitor, influence, and manipulate the microbial community, thereby impacting pathogen transmission) within these communities, crucial for manipulating tick microbiomes toward disease control, remains inadequate. To address these challenges, we collected 4,167 ticks during 2021–2022 and performed morphological assessments. We selected representative samples and grouped them by six factors for full-length 16S rRNA sequencing to analyze microbial community characteristics and differences. We also tested for seven common tick-borne pathogens and conducted IgG testing in Hainan Island residents. Our aim is to evaluate the characteristics and impacts of tick microbial communities under these six factors in Hainan Island. By assessing pathogen positivity rates corresponding to these factors, we infer their influence on tick vectorial competence. Furthermore, we identify potential biomarkers within the microbial communities that could serve as targets for local tick and tick-borne disease control strategies.

## Methods

### Sample collection and processing

**Figure 1:**
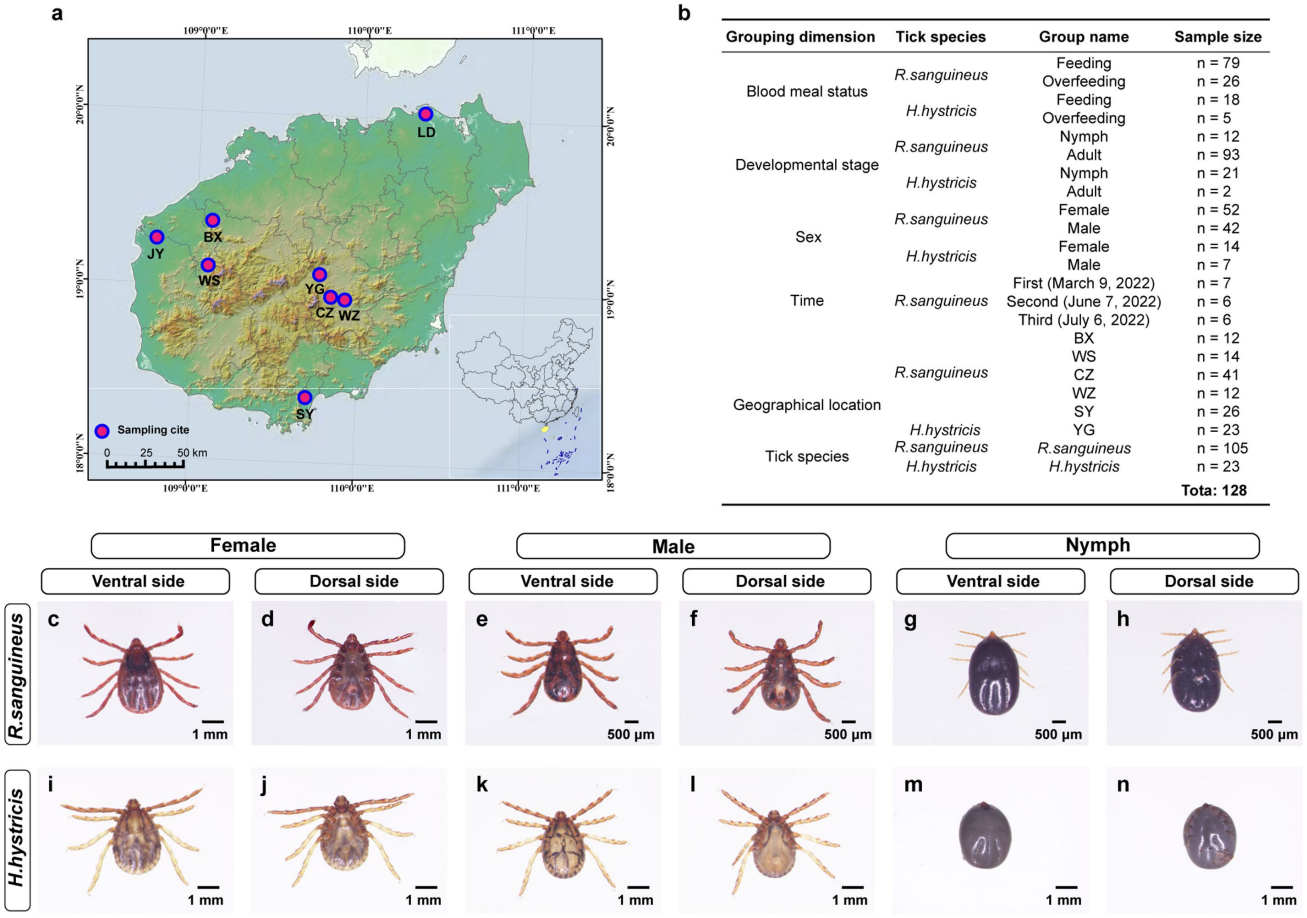
Tick sampling locations and morphological classification on Hainan Island. Panel **(a)** illustrates Hainan Island’s tick sampling sites at Lindan (LD), Jiangyuan (JY), Bangxi (BX), Wushi (WS), Yinggen (YG), Changzhen (CZ), Wanzhong (WZ), and Sanya (SY), marked with pink circles. Panel **(b)** categorizes 128 tick samples by blood meal status, developmental stage, sex, time, geographical location, and tick species, for *R. sanguineus* and *H. hystricis*, with subgroup details and sample counts. Panels **(c–n)** display the morphologies of *R. sanguineus* and *H. hystricis* across different sexes and life stages (female: **c,d,i,j**; male: **e,f,k,l**; nymph: **g,h,m,n**), offering ventral and dorsal views to highlight species-specific traits.

Between 2021 and 2022, we collected 4,167 ticks from dogs and goats across Hainan Island, China (Figure 1a; Supplementary Table S1). Of these, approximately 96 were identified as *H. hystricis*, with the majority of the remaining ticks classified as *R. sanguineus*. All samples were transported to the laboratory on the same day and stored at-80°C until further processing. Morphological characteristics were identified based on previous studies (Tanskul and Inlao, 1989; Walker et al., 2000; Estrada-Peña, 2004), and species were confirmed by amplifying the *COXI* gene (Chitimia et al., 2010) (Supplementary File S1). Specifically, Sample processing involved cleaning ticks with 75% ethanol and a brush (repeated twice), and observing under a stereomicroscope to determine species, developmental stage, sex, and blood meal status (Figure 1b). Ticks were then ground in Trizol using a tissue grinder, and nucleic acids were extracted following methods from previous studies (Yu et al., 2022). DNA quality and concentration were assessed using a microplate reader (SYNERGY H1), and nuclease-free water was used as a blank control during each DNA extraction to ensure no contamination. Samples with concentrations below 20 ng/μl were excluded. The total DNA extracted from ticks was used for sequencing and pathogen detection.

To explore the impact of various factors on tick microbial communities, we grouped the samples based on blood meal status (completely engorged ticks were defined as overfeeding, while others were defined as feeding), developmental stage (based on morphological characteristics), sex (based on morphological characteristics), time (based on sampling date), geographical location (based on sampling site), and tick species (based on morphological characteristics and COXI gene identification) (Figure 1b; Supplementary Table S2). We selected 128 samples for 16S rRNA sequencing and 772 samples for pathogen detection, based on these grouping dimensions. This selection aimed to balance representativeness across different groups while managing available resources and ensuring sufficient data quality for reliable analysis.

In February 2024, we collected 208 residual clinical blood samples from Hainan Medical University’s Second Affiliated Hospital for tick-borne pathogen IgG antibody testing, with hospital approval.

### Microbial sequencing analysis

We amplified full-length 16S rRNA sequences using Phusion High-Fidelity PCR Master Mix with GC Buffer (New England Biolabs) with primers F: 5‘-AGAGTTTGATCCTGGCTCAG-3’ and R: 5‘-GNTACCTTGTTACGACTT-3’. Library construction and sequencing on the PacBio platform were performed by Novogene (China). To control for potential contamination during the sequencing process, nuclease-free water was used as a negative control. Downstream analysis was conducted on 128 quality-checked and filtered samples, with reads standardized to a maximum sequencing depth of 25,608 reads per sample (Supplementary Table S2). All raw data were processed in R using the DADA2 package (Callahan et al., 2016). These were integrated into a Phyloseq object for downstream analysis (Mcmurdie and Holmes, 2013). Relative abundance bar plots and heatmaps were generated using Phyloseq. Alpha diversity metrics (observed, Shannon, Pielou) and beta diversity analyses using Principal Coordinates Analysis (PCoA) based on Bray-Curtis distance (vegan package) were calculated. Differential species analysis was performed using LEfSe (threshold: *p* < 0.05, LDA > 2.0), Venn (*p* < 0.05), ALDEX2 (*p* < 0.05), and RandomForest methods. Robust differential species were identified by the intersection of these four methods. Functional predictions for each microbial community were conducted using PICRUST2. Functional abundance and differential function analyses were performed at level 3, with functional PCoA metrics based on Bray-Curtis distances. Robust differential functions were determined by the intersection of Venn, RandomForest, and ALDEX2 results. Microbial communities were classified into transient and stable species based on an occurrence frequency threshold of ≥50%, following B Glasl et al. (2019). The same parameters were used for species-level and function-level analyses of stable and transient species. Microbial networks were constructed based on Spearman’s correlation and visualized using Gephi (0.10). Network nodes were categorized by within-module connectivity (Zi) and among-module connectivity (Pi) values into four types: Module hubs (Zi > 2.5, Pi <0.62), Connectors (Zi < 2.5, Pi >0.62), Network hubs (Zi > 2.5, Pi >0.62), and Peripherals (Zi < 2.5, Pi <0.62) (Guimera and Nunes Amaral, 2005). Nodes resulting in NAs due to transient species were also considered. Robust core species were determined by the intersection of stable species, high-abundance species (abundance >0.1%), and top 100 network topology parameters (Degree, Closeness, Betweenness, Eigenvector). Visualizations were performed using the ggplot2 package in R (Wickham et al., 2019).

### Pathogen nucleic acid and IgG antibody detection

Genomic DNA extracted from tick samples was used to detect seven common tick-borne pathogens, including *Borrelia burgdorferi*, *Rickettsia* spp., *Ehrlichia* spp., *Anaplasma* spp., *Babesia* spp., *Theileria* spp., and *Hepatozoon* spp. Each pathogen gene fragment was amplified in a 25 μL reaction mixture containing 12.5 μL Polymerases (RR371, TaKaRa, Japan), 1 μL forward primer (10 μM), 1 μL reverse primer (10 μM), 1 μL DNA template, and DNase/RNase-free distilled water (TaKaRa, Japan). Primer sequences and sources are listed in Supplementary Table S3 (Otranto et al., 2011; Chaisi et al., 2017; Levytska et al., 2021; Zhang et al., 2022; Norouzi et al., 2023). Touchdown PCR was performed based on the primer Tm values (Korbie and Mattick, 2008). DNase/RNase-free distilled water was used as the negative control. PCR products were electrophoresed on 1.5% agarose gels and visualized using a gel imaging system. Positive samples were validated by unidirectional Sanger sequencing (Sangong Biotech). Serum samples were tested for *Rickettsia* and *Borrelia burgdorferi s.l.* IgG using commercial kits (mlbio, China) according to the manufacturer’s protocol, which includes positive and negative controls. The absorbance threshold for positive samples was defined as per the kit instructions.

### Statistical analysis

After standardizing sequencing depth across all samples, analyses were performed based on the normalized ASV absolute count matrix. Alpha diversity differences between groups were analyzed using the Mann–Whitney test (for two-group comparisons: sex, developmental stage, tick species, and blood meal status) and the Kruskal-Wallis test (for multi-group comparisons: time and geographical location), with Dunn’s test for *post hoc* multiple comparisons. Beta diversity was assessed using Bray-Curtis distance and PCoA clustering. PERMANOVA was employed to test for significant differences in overall microbial community structure across the six previously mentioned grouping dimensions, while PERMDISP was used to evaluate the homogeneity of multivariate dispersion within each group. For Venn analysis of species, a threshold of 0.5 was used to reduce sequencing errors and individual variation, identifying unique and shared species through subset intersections. Differential abundance analysis, as well as comparisons of the number and abundance of transient and stable species, were conducted using the Mann–Whitney test (for two-group comparisons) and the Kruskal-Wallis test (for multi-group comparisons), with Dunn’s test for *post hoc* analysis. Groupings and samples considered in this analysis were consistent with those used in Alpha and Beta diversity analyses. Species-level and function-level PCoA analyses used PERMANOVA. Fisher’s exact test was used to compare pathogen positivity rates and IgG positivity rates among different groups. *p*- values less than 0.05 were considered statistically significant.

## Results

In this study, we selected 128 whole tick samples from 4,167 samples for full-length 16S rRNA sequencing of microbial communities (Figure 1b). The datasets are available in the SRA repository under BioProject ID PRJNA1073713. Additionally, 772 samples were selected for pathogen detection of *Borrelia burgdorferi*, *Rickettsia* spp., *Ehrlichia* spp., *Anaplasma* spp., *Babesia* spp., *Theileria* spp., and *Hepatozoon* spp. The sample selection was based on tick species and sex, developmental stage, blood-feeding status, sampling time, geographical location, and other relevant factors. These samples represent a broad diversity across various grouping dimensions, ensuring that we could thoroughly explore key changes in microbial communities.

### Microbial community structure

**Figure 2:**
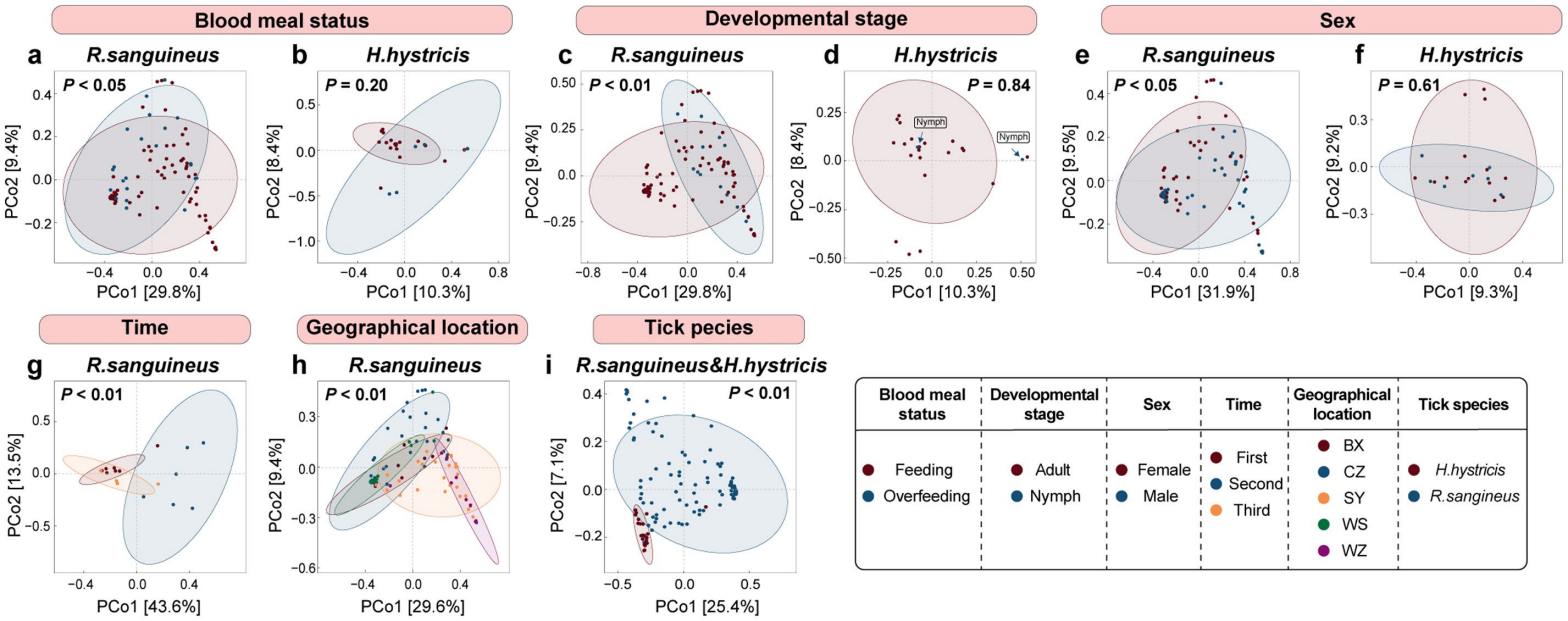
Species-level microbial diversity and alpha diversity in *R. sanguineus* and *H. hystricis*. Panels **(a–i)** depict a PCoA of the beta diversity at the species level in the microbiomes of *R. sanguineus* and *H. hystricis*, based on Bray-Curtis distances. The percentages on the axes indicate the variance explained. Confidence ellipses provide a 95% certainty level for the centroids of each group, with PERMANOVA *p*-values noted. The panels detail: blood meal status in *R. sanguineus* (a) and *H. hystricis* **(b)**; sex and developmental stages in *R. sanguineus* **(c,e)** and *H. hystricis* **(d,f)**; along with temporal **(g)** and regional **(h)** variations in *R. sanguineus*, and interspecies comparisons **(i)**. PERMDISP was also used to explore the contribution of within-group dispersion to these community differences. Detailed results for both PERMANOVA and PERMDISP analyses are provided in Supplementary Table S4.

**Figure 3:**
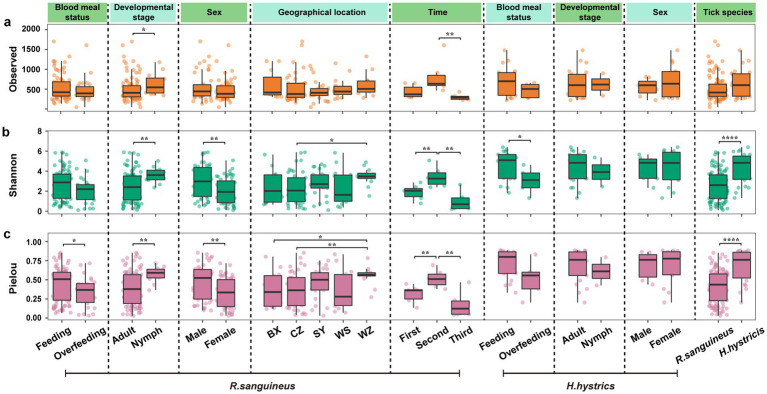
Aalpha diversity in *R. sanguineus* and *H. hystricis*. Panels **(a–c)** present Alpha diversity metrics for both tick species. Panel **(a)** shows observed species richness, Panel **(b)** shows Shannon diversity, and Panel **(c)** shows Pielou’s evenness. The metrics are categorized by factors like blood meal status, developmental stage, sex, time, geographical location, and tick species. Dashed and solid lines distinguish between factors and species, respectively. Significance levels are marked by asterisks (*p* < 0.05; ** *p* < 0.01; *** *p* < 0.001; **** *p* < 0.0001), with detailed statistics in Supplementary Table S4, including pairwise comparisons for ‘geographical location’ and ‘time’.

At the overall microbial species level, we used Bray-Curtis distance-based PCoA to describe the microbial communities (beta diversity). The results showed that for *R. sanguineus*, factors such as blood meal status (*n* = 79 for Feeding, *n* = 26 for Overfeeding, Figure 2a), developmental stage (*n* = 12 for Nymph, *n* = 93 for Adult, Figure 2c), sex (*n* = 52 for Female, *n* = 42 for Male, Figure 2e), time (*n* = 7 for First, *n* = 6 for Second, *n* = 6 for Third, Figure 2g), and geographical location (*n* = 12 for BX, *n* = 14 for WS, *n* = 41 for CZ, *n* = 12 for WZ, *n* = 26 for SY, Figure 2h) all had statistically significant effects on microbial communities, as indicated by PERMANOVA (*p* < 0.05). This suggests that these factors influence the microbial communities of *R. sanguineus*. However, PERMDISP analysis suggested that the observed effects of time and geographical location on the microbial communities of *R. sanguineus* may be due to variations in community dispersion within the populations. Further comparisons indicated that statistically significant differences in dispersion were present only between specific time points (‘second-third’, *F* = 5.76, *p* < 0.05) and locations (‘BX-SY’, *F* = 9.91, *p* < 0.05; ‘CZ-SY’, *F* = 8.55, *p* < 0.01; ‘SY-WS’, *F* = 9.92, *p* < 0.01; Supplementary Table S4). For *H. hystricis*, although the PCoA plots showed clustering patterns similar to those of *R. sanguineus* across grouping dimensions such as blood meal status (*n* = 18 for Feeding, *n* = 5 for Overfeeding, Figure 2b), developmental stage (*n* = 21 for Nymph, *n* = 2 for Adult, Figure 2d), and sex (*n* = 14 for Female, *n* = 7 for Male, Figure 2f), suggesting similar effects on microbial communities, PERMANOVA did not reveal significant differences (Figures 2b,d,f; Supplementary Table S4). This lack of significance may be due to insufficient sample size or the absence of true differences. However, PERMDISP revealed significant differences in dispersion associated with blood meal status (*F* = 34.33, *p* < 0.001) and nymph-adult stages (*F* = 62.91, *p* < 0.001), despite PERMANOVA showing no such differences. This suggests that *H. hystricis* displays greater individual variability compared to *R. sanguineus*, while overall community composition remains similar. The significant differences detected by both PERMANOVA and PERMDISP in the tick species grouping dimension further confirm this observation (*n* = 105 for *R. sanguineu*, *n* = 23 for *H. hystricis*, Figure 2i). Alpha diversity metrics further revealed the specific impacts of these factors on microbial communities. In *R. sanguineus*, the number of observed species significantly increased in adults (Figure 3a), with the highest observed index at the second time point (Figure 3a). This pattern was not observed in *H. hystricis*. Additionally, there was no significant difference in the observed index between the two tick species. The Shannon index, which represents microbial community diversity, was higher in adult *R. sanguineus* compared to nymphs, higher in males compared to females, and highest at the second time point compared to the other two time points. For *H. hystricis*, the Shannon index was significantly higher before overfeeding compared to after overfeeding (*p* < 0.05), a difference not seen in *R. sanguineus*. This reflects species differences, confirmed by the significant differences in Shannon index comparisons in the tick species grouping dimension (Figure 3b). The Pielou index explains the differences in microbial community evenness. In *R. sanguineus*, evenness was higher before overfeeding, higher in nymphs compared to adults, and higher in males compared to females (Figure 3c). There were also differences in microbial community evenness among different geographical locations, with the second time point showing the highest evenness. *H. hystricis* had higher evenness than *R. sanguineus* (Figure 3c).

We observed that all three alpha diversity parameters were higher at the second time point compared to the other two time points (Figures 3j–l), highlighting the uniqueness of this time point and aligning with the temporal separation seen in the PCoA (Figure 2g). This suggests temporal fluctuations and inconsistencies in microbial communities. Additionally, there were differences among geographical locations; some regions showed significant differences while others did not (Figures 3a–c). The clustering patterns in the PCoA also reflected both similarities and differences between regions (Figure 2h). The pairwise comparisons for geographical and temporal dimensions can be found in Supplementary Table S4.

At the phylum level, the overall microbial community was dominated by Firmicutes and Proteobacteria (Supplementary Figure S1a). At the genus level, the main taxa were *Macrococcus*, *Staphylococcus*, and *Coxiella* (Supplementary Figure S1c). Differences between subgroups were mainly observed in the subdominant species. High-abundance species, such as *Macrococcus*, *Staphylococcus*, and *Coxiella*, were generally stable species. However, some low-abundance species, such as *Corynebacterium* and *Delftia*, were also stable in certain groups, and there was a notable shift between transient and stable species, underscoring the importance of low-abundance and transient species (Supplementary Figure S1d).

### Microbial community function

**Figure 4:**
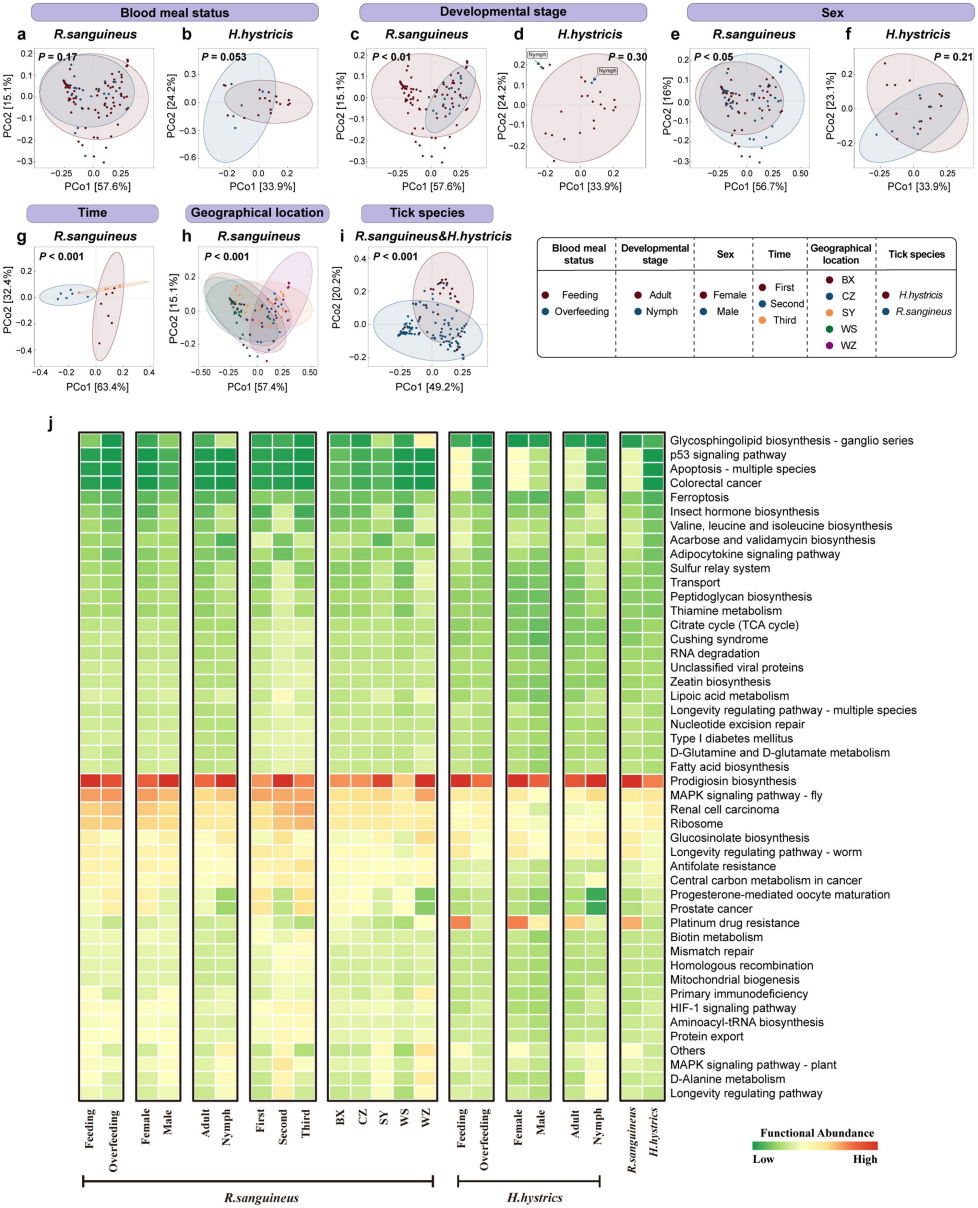
Overall functional-level PCoA analysis and heatmap for *R. sanguineus* and *H. hystricis*. Panel **(a–i)** depict PCoA plots illustrating functional beta diversity, with PERMANOVA p-values signifying significance. Panels **(a)** and **(b)** show blood meal status-related diversity in *R. sanguineus* and *H. hystricis*. Panels **(c)** and **(d)** address developmental stage within each species. Panels **(e)** and **(f)** cover sex diversity within each species, respectively. Panels **(g)** and **(h)** show temporal and regional diversity in *R. sanguineus*. Panel **(i)** facilitates a functional comparison between the two species. Ellipses represent confidence intervals, accompanied by a color legend. Panel **(j)** depicts a heatmap that ranks functional abundance from low (green) to high (red) for *R. sanguineus* (blood meal status, developmental stage, sex, time, geographical location, and tick species) and *H. hystricis* (blood meal status, developmental stage, sex), alongside a cross-species functional comparison. Supplementary Table S4 contains detailed PERMANOVA statistics, including pairwise comparisons for the ‘geographical location’ and ‘time’ dimensions, as well as the results from PERMDISP analysis.

Using PICRUST2 for functional analysis, PCoA based on Bray-Curtis distance at the overall functional level of microbial communities showed statistically significant differences in *R. sanguineus* across different developmental stages (Figure 4c), sex (Figure 4e), time points (Figure 4g), and geographical locations (Figure 4h) (PERMANOVA, *p* < 0.05). There were also significant differences between *R. sanguineus* and *H. hystricis* (Figure 4i). However, no statistical differences were observed in *R. sanguineus* before and after overfeeding, nor in *H. hystricis* before and after overfeeding, between sex, or across different developmental stages. The PERMDISP results indicated that functional level dispersion differences were only observed between *R. sanguineus* nymphs and adults, while no significant differences in dispersion were found among the other groupings of *R. sanguineus* and *H. hystricis* (Supplementary Table S4).

A heatmap of functional abundances based on PICRUST2 indicated that the Prodigiosin biosynthesis pathway dominated in both tick species across all groups (Figure 4j). Additionally, differences in less dominant functions were observed between groups, such as the Progesterone-mediated oocyte maturation pathway, the Prostate cancer pathway, and the Platinum drug resistance pathway. PICRUST2 functional predictions revealed differences in key functional pathways among different groups of microbial communities. However, these predictions have not been extensively validated in ticks, and errors may exist. Future studies should experimentally verify the actual presence and biological significance of these functional pathways to gain a more accurate understanding of the functional characteristics of tick microbial communities.

### Differential analysis at species and functional levels

The Venn analysis revealed the unique and shared species within microbial communities under different conditions. Overall, in both *R. sanguineus* and *H. hystricis*, the number of microbial community species decreased after overfeeding at the overall level (Supplementary Figures S2a1,a2), stable species level (Supplementary Figures S2b1,b2), and transient species level (Supplementary Figures S2c1,c2). However, at the functional level, the two tick species showed different patterns (Supplementary Figures S2d1,d2).At different developmental stages, the trends varied between the two tick species. Nymphs of *R. sanguineus* had more unique species and unique stable species (Supplementary Figures S2a3,b3), whereas *H. hystricis* showed the opposite trend (Supplementary Figures S2a4,b4). Between sex, males of both *R. sanguineus* and *H. hystricis* had more unique species and unique stable species (Supplementary Figures S2a5,a6,b5,b6). However, *R. sanguineus* males had more unique transient species, whereas *H. hystricis* males showed the opposite trend (Supplementary Figures S2c5,c6). Compared to *H. hystricis*, *R. sanguineus* had more unique species, unique stable species, and unique transient species. At the functional level, *R. sanguineus* did not exhibit unique functions, indicating that *H. hystricis* has more functional uniqueness (Supplementary Figures S2a7,b7,c7,d7). In the temporal dimension, the first time point had more unique species, unique stable species, and unique transient species (Supplementary Figures S2a8,b8,c7). In the upset analysis across different geographical locations, the WZ region had the most unique species and unique stable species (Supplementary Figures S2a9,b9), while the CZ region had the most unique transient species (Supplementary Figure S2c9). Detailed lists of these species can be found in Supplementary File S2.

Furthermore, we used Aldex2, Lefse, RandomForest, and Venn (unique species + shared species with statistically significant abundance differences) methods to identify differential species, intersecting these to obtain robust differential species (Supplementary Figures S3a–q), and generated corresponding lists (Supplementary Table S5). Detailed lists of differential species and differential functions in each intersection can be found in Supplementary Files S3, S4.

### Transient and stable species in microbial communities

**Figure 5:**
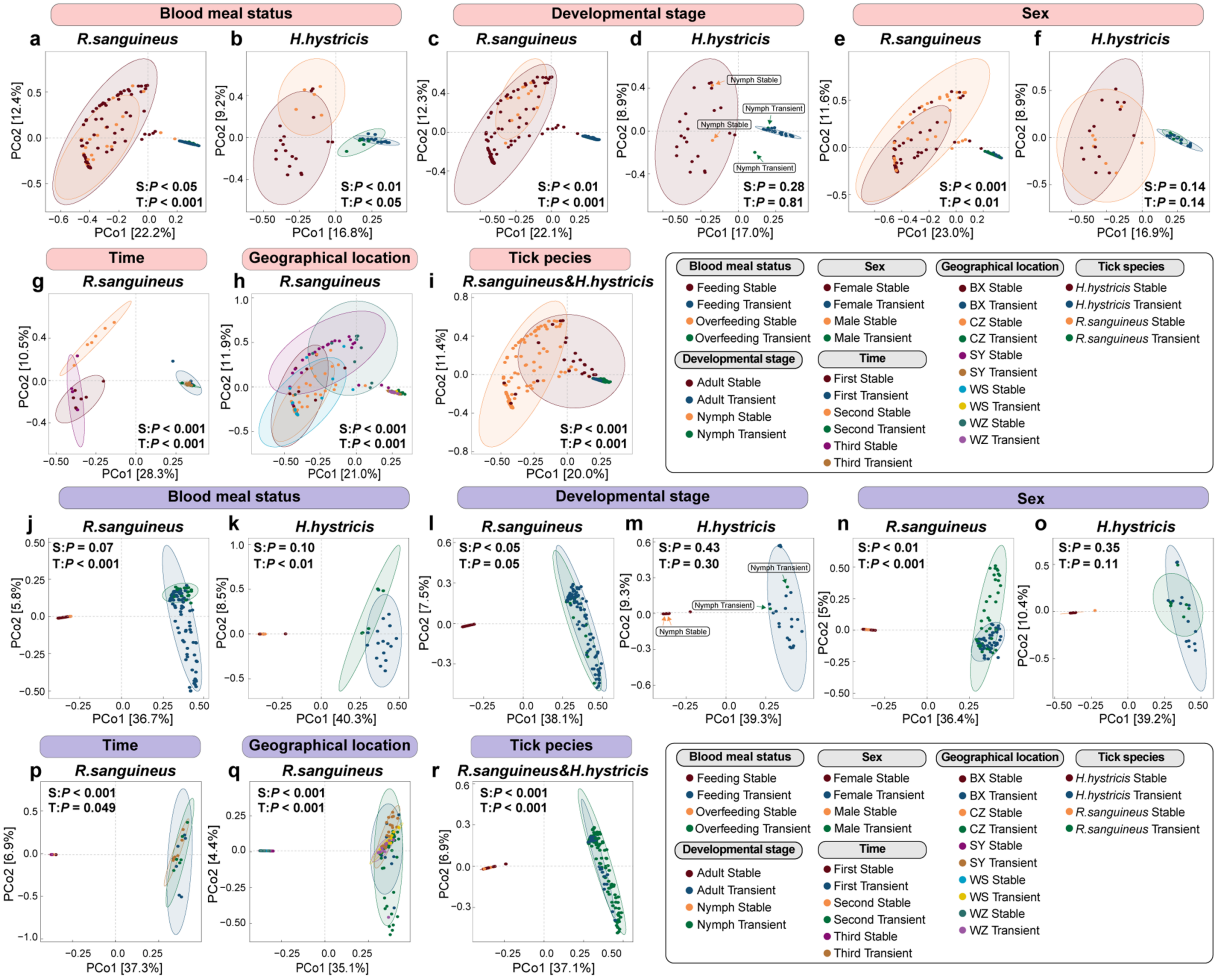
Microbial community partition analysis of transient versus stable species by PCoA at species and functional levels. Species-level PCoA plots (panels **a–i** with pink labels) delve into the diversity within stable and transient species, while functional-level PCoA plots (panels **j–r** with purple labels) evaluate functional diversity. These plots employ Bray–Curtis dissimilarity measures and feature PERMANOVA *p*-values, denoting “S:” for stable and “T:” for transient species comparisons. Supplementary Figure S4 provides individual PCoA plots for each species type to enhance clarity obscured in combined plots. Comprehensive statistical analyses, including PERMANOVA and PERMDISP results with pairwise comparisons for ‘geographical location’ and ‘time’, are accessible in Supplementary Table S4.

To further explore how different species in tick microbial communities respond to these factors, we divided the microbial communities into stable species and transient species based on their frequency of occurrence. We conducted PCoA analysis using Bray-Curtis distance and detected differences through PERMANOVA at both the species level (Figures 5a–i) and the functional level (Figures 5j–r). At the species level in the microbial communities of *R. sanguineus*, both stable and transient species contributed to changes in microbial communities across blood meal status, developmental stages, sex, time, and geographical locations (Figures 5a,c,e,g,h). PERMDISP results showed that except for significant differences in transient species dispersion across blood meal status in *R. sanguineus* (*F* = 6.22, *p* < 0.05), no other grouping dimensions exhibited changes in dispersion for either stable or transient species (Supplementary Table S4). *H. hystricis* showed joint contributions from stable and transient species only after overfeeding (Figure 5b). Unlike *R. sanguineus*, PERMDISP revealed differences in transient species dispersion between nymph and adult stages in *H. hystricis*, while PERMANOVA did not find differences in their composition. The results from both PERMANOVA and PERMDISP indicate that stable and transient species contributed to microbial community changes in *R. sanguineus* and *H. hystricis* under various factors. For certain factors, such as transient species under the blood meal status in *R. sanguineus* and both stable and transient species under the tick species grouping dimension, these changes may be driven by variations in within-group dispersion (Supplementary Table S4). These findings indicate that, beyond differences in the composition and dispersion of stable and transient species between the two tick species, transient species also play a key role in shaping microbial communities, and their replacement is non-random.To better illustrate the relationship between stable and transient species communities, we visualized them separately in PCoA plots (Supplementary Figure S4).

At the functional level, PERMANOVA indicated that three response patterns emerged: stable species-driven, transient species-driven, and jointly driven by both stable and transient species. For example, the transition of *R. sanguineus* from nymph to adult was driven solely by stable species (Figure 5i). However, functional changes in both *R. sanguineus* and *H. hystricis* after overfeeding were driven only by transient species (Figures 5j,k), highlighting the importance of transient species. PERMDISP analysis similarly confirmed that these changes were not due to variations in within-group dispersion (Supplementary Table S4). Changes in microbial communities in terms of sex, time, geographical location, and tick species were driven by both stable and transient species (Figures 5n–r). The functional responses of stable and transient species were visualized through PCoA (Supplementary Figure S4).

**Figure 6:**
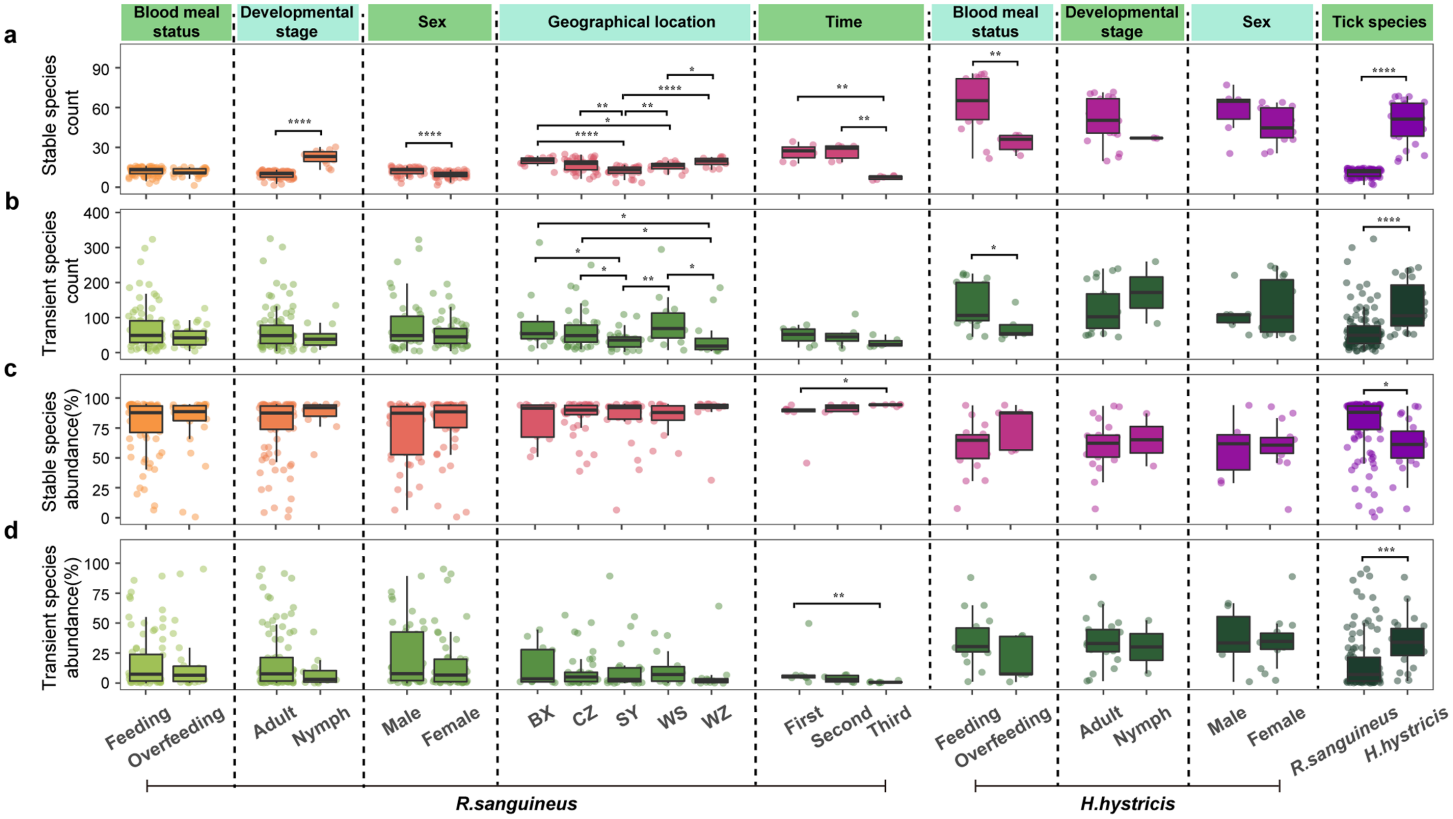
Microbial community partition analysis of transient versus stable species by count and abundance. Panels **(a–d)** segment tick microbiomes into transient and stable categories, subsequently analyzing their count (**a** for stable, **b** for transient) and total abundances (**c** for stable, **d** for transient) across six dimensions for *R. sanguineus* and *H. hystricis*. Dashed lines delineate the dimensions. Comprehensive statistical analyses, including PERMANOVA and PERMDISP results with pairwise comparisons for ‘geographical location’ and ‘time’, are accessible in Supplementary Table S4.

In all groups, the number of transient species was much higher than that of stable species, but stable species accounted for a higher proportion of relative abundance (Figures 6a–d). The six factors induced different response patterns in terms of species numbers and relative abundance of stable and transient species. Specifically, *R. sanguineus* nymphs had more stable species compared to adults (Figure 6a), and males had more stable species compared to females (Figure 6a). Changes in transient and stable species between different geographical locations were mainly reflected in species numbers, and not all regions showed differences (Figures 6a,b). Temporally, the response patterns of stable and transient species varied across different time points, with not all time points showing changes, which was similarly reflected in geographical dimensions (Figures 6a,b). After overfeeding, the number of stable and transient species did not change in *R. sanguineus*, but both decreased in *H. hystricis* (Figures 6a,b), highlighting species-specific differences. The comprehensive differences in species numbers and relative abundance of stable and transient species between the two tick species further confirmed this (Figures 6a–d).

### Microbial co-occurrence networks

**Figure 7:**
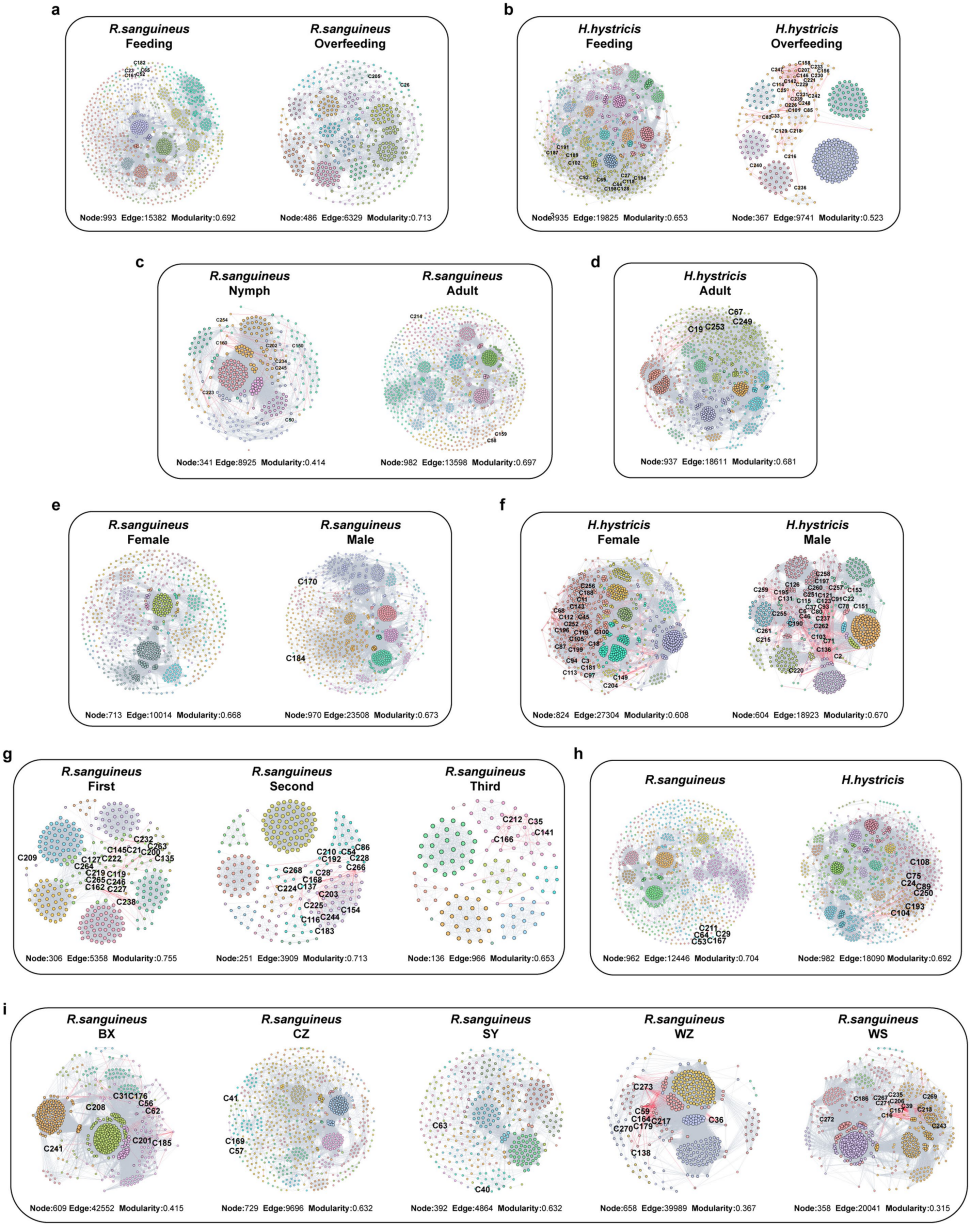
Six-dimensional microbial network analysis in *R. sanguineus* and *H. hystricis*. This figure delineates the microbial interaction networks within two tick species across various dimensions: blood meal status [*R. sanguineus*: **(a)**; *H. hystricis*: **(b)**], developmental stages [*R. sanguineus*: **(c)**; *H. hystricis*: **(d)**], sex [*R. sanguineus*: **(e)**; *H. hystricis*: **(f)**], time **(g)**, species comparison **(h)**, and geographical location **(i)**. Nodes reflect species, sized by relative abundance, with network edges in gray or red indicating positive or negative interactions, respectively. Further details on network topology, stability, and node classification are provided in Figure 8, while counts of core species for each group are presented in Graphical abstract. Detailed information on robust core species is listed in Supplementary File S6. These networks underscore the complex relationships and ecological niches within tick microbiomes, considering various life history traits and environmental contexts.

**Figure 8:**
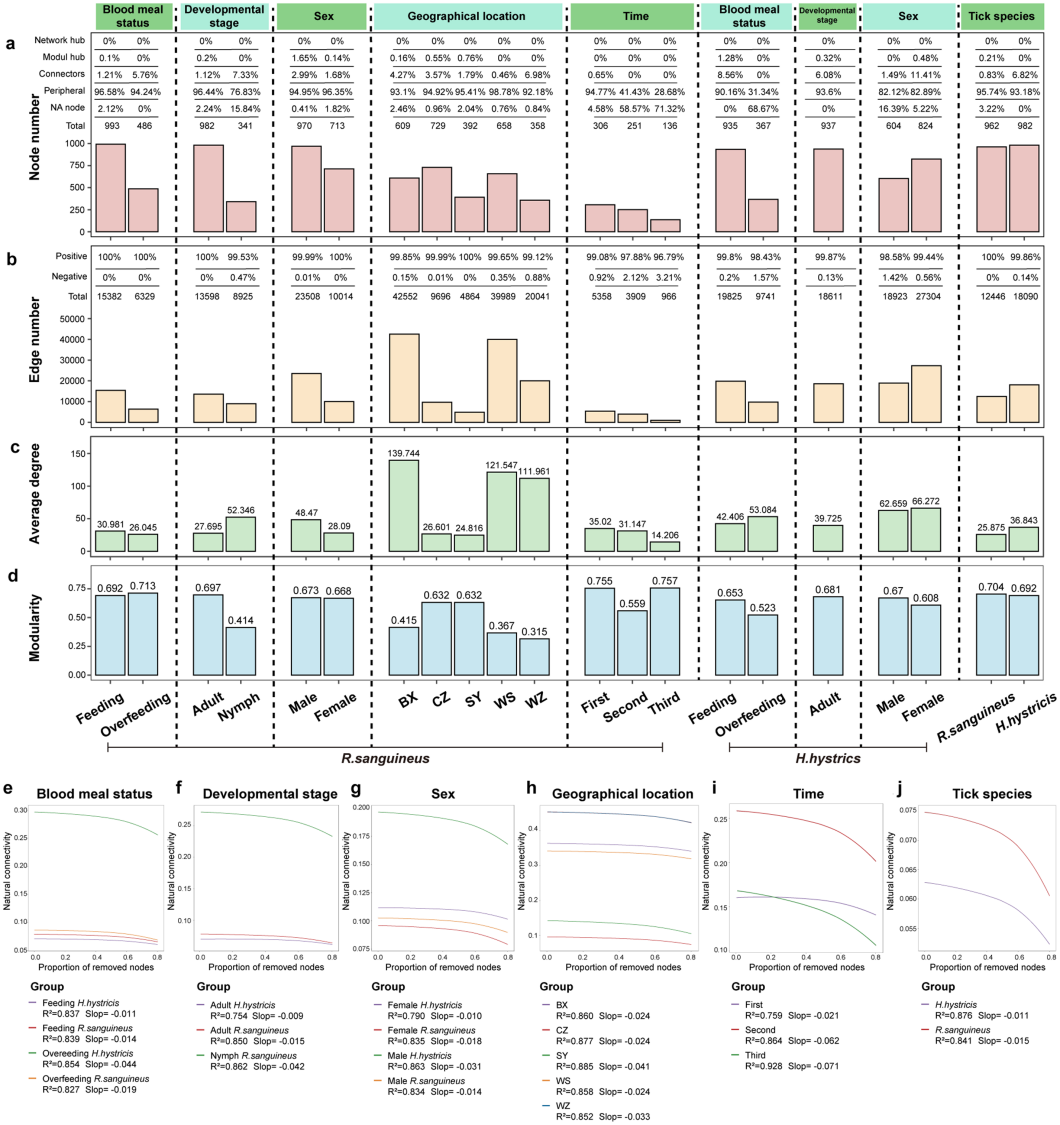
Network topology and stability analysis of tick-associated microbial communities. Panel **(a)** enumerates nodes within tick-associated microbial communities, categorizing them into network hubs, module hubs, connectors, peripherals, and NA nodes based on their within-module (Zi) and among-module (Pi) connectivity: module hubs (Zi > 2.5, Pi <0.62), connectors (Zi < 2.5, Pi >0.62), network hubs (Zi > 2.5, Pi >0.62), and peripherals (Zi < 2.5, Pi <0.62). NA nodes, not fitting into these classifications, may represent transient species. Panels **(b–d)** illustrate the edge count, average degree, and modularity, across various groupings: blood meal status, developmental stage, sex, time, geographical location, and tick species. Panels **(e–j)** display natural connectivity curves for these subgroups, where the y-axis denotes network stability (natural connectivity) and the x-axis shows the cumulative number of nodes removed. A higher initial y-axis value suggests greater network stability, with a steeper slope indicating reduced resilience. R-squared values in the curves reflect the model’s capacity to explain each subgroup’s stability resilience.

To illustrate the impact of different factors on microbial community interactions and stability, we constructed microbial co-occurrence networks for various groups (Figures 7a–i). Additionally, information on microbial network nodes and edges is provided in Supplementary File S5. After overfeeding, *R. sanguineus* showed a decrease in both the number of network nodes and edges, indicating a reduction in diversity and interactions due to overfeeding. This phenomenon was also observed in *H. hystricis* (Figures 8a,b). However, the trends in average degree and modularity of *R. sanguineus* after overfeeding were opposite to those in *H. hystricis* (Figures 8c,d). As *R. sanguineus* transitioned from nymph to adult, both node and edge numbers increased, while the average degree decreased and modularity increased (Figures 8a–d). Due to insufficient samples, we could not construct a network for *H. hystricis* nymphs (Figures 8a,b). In terms of sex, male *R. sanguineus* displayed higher node number, edge number, and average degree, whereas in *H. hystricis*, females showed higher values for these metrics (Figures 8a–c). There were significant differences in the microbial co-occurrence networks of *R. sanguineus* across different geographical locations and time points (Figures 8a–d). Although *H. hystricis* had a similar number of nodes compared to *R. sanguineus*, it exhibited higher edge numbers and average degree (Figures 8a–d).

Furthermore, we classified the nodes based on Zi and Pi values, finding that peripheral nodes dominated the microbial co-occurrence networks across all groups (Figure 8a). This suggests that the network structures are relatively loose, likely due to the dominance of transient species in the tick microbial communities. To identify core species in each state, we selected the top 100 species based on four topological parameters, intersecting these with high-abundance and stable species to determine robust core species (Supplementary Figure S5; Supplementary Table S5; Supplementary File S6).

The natural connectivity curves of microbial networks, representing network stability and resilience to disturbances, showed initial advantages for overfed *H. hystricis*, nymph *R. sanguineus*, male *H. hystricis*, the BX region, the second time point, and *R. sanguineus* in their respective groups. However, the decrease in natural connectivity was relatively gradual, possibly due to the predominance of peripheral nodes (Figures 8e–j).

### Tick-borne pathogen detection

To clarify the current status of tick-borne diseases in Hainan Island and to support the potential link between microbial community changes and vectorial capacity, we tested 772 samples for seven common tick-borne pathogens. We detected six genera and eight species of tick-borne pathogens in 12.44% (96/772) of the samples (Table 1), but *B. burgdorferi* was not found. Among these 96 positive samples, 22.92% carried two or more pathogens. Differences in the positivity rates of *Rickettsia*, *Ehrlichia*, *Theileria*, and *Hepatozoon* under different conditions suggest that these six factors might influence the vectorial capacity of ticks (Supplementary Table S6). However, no statistical differences in the positivity rates of *Anaplasma* and Babesia were observed across these six factors, suggesting that these factors might not affect the vectorial capacity of ticks for these two pathogens (Supplementary Table S6). Although further verification is needed, these findings broadly indicate that different pathogens may prefer ticks in different states.

**Table 1:** Detection and co-infection rates of tick-borne pathogens.

Pathogens/Combination	Positive number	Total
Detected pathogens		
*Rickettsia montanensis*	1/772(0.13%)	96/772 (12.44%)
*Rickettsia rhipicephali*	3/772 (0.39%)	
*Borrelia burdorferi* s.l.	0/772 (0%)	
*Ehrlichia canis*	49/772 (6.35%)	
*Anaplasma platys*	8/772 (1.04%)	
*Babesia vogeli*	9/772 (1.17%)	
*Theileria sinensis*	1/772 (0.13%)	
*Theileria luwenshuni*	1/772 (0.13%)	
*Hepatozoon canis*	50/772 (6.48%)	
Co-infection types		
*Rickettsia*	4/96 (4.17%)	96/96 (100%)
*Ehrlichia*	31/96 (32.29%)	
*Anaplasma*	5/96 (5.21%)	
*Babesia*	3/96 (3.13%)	
*Theileria*	2/96 (2.08%)	
*Hepatozoon*	29/96 (30.21%)	
*Ehrlichia* + *Hepatozoon*	16/96 (16.67%)	
*Anaplasma* + *Hepatozoon*	2/96 (2.08%)	
*Anaplasma* + *Babesia*	1/96 (1.04%)	
*Babesia* + *Hepatozoon*	1/96 (1.04%)	
*Ehrlichia* + *Babesia* + *Hepatozoon*	2/96 (2.08%)	

This table includes the detection rates of seven common tick-borne pathogens and the types of co-infection combinations. The “Detected pathogens” section shows the detection rates for seven specific pathogen species, with the positive rates calculated using a total of 772 tick samples. The “Co-infection types” section presents the detection rates for various co-infection combinations, including single pathogen infection types, with the positive rates calculated using the 96 samples that tested positive for at least one pathogen.

**Figure 9:**
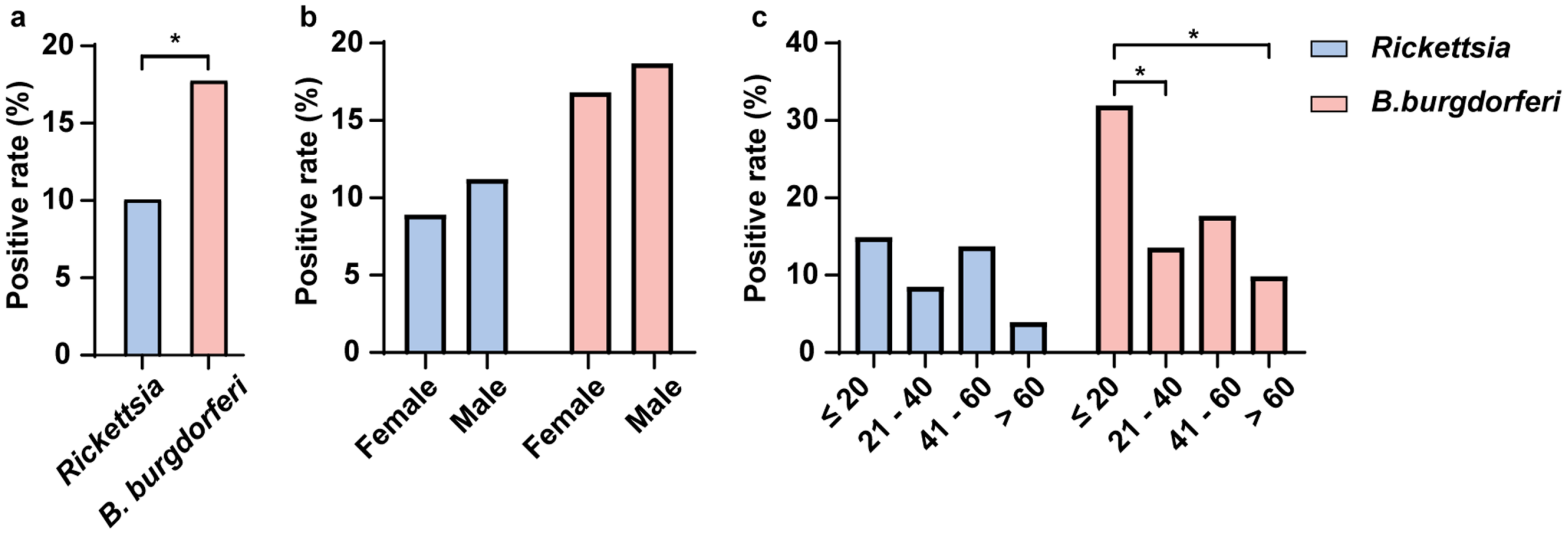
Detection of *Rickettsia* and *B. burgdorferi* IgG Antibodies in the Serum of Residents of Hainan Island. This figure illustrates the seroprevalence of IgG antibodies specific to two pathogens (*Rickettsia* and *B. burgdorferi*) in human serum samples. Panel **(a)** shows the overall positivity rates of both pathogens in the population. Panel **(b)** displays the positivity rates of each pathogen across different sex. Panel **(c)** presents the positivity rates of both pathogens across different age groups. All positivity rates were statistically analyzed using Fisher’s exact test, and *p*- values are indicated in the figure (* *p* < 0.05). Different colors represent different pathogens.

Additionally, we tested the sera of 208 residents of Hainan Island for IgG antibodies against *Rickettsia* and *B. burgdorferi*. The results showed that 18.27% (38/208) of the sera samples were positive for at least one tick-borne pathogen IgG, with 170 samples being negative for both. The overall IgG positivity rate for *Rickettsia* was 10.10% (21/208), and for *B. burgdorferi* s.l., it was 17.79% (37/208), with 9.13% (19/208) of the samples positive for both *Rickettsia* spp. and *B. burgdorferi* s.l. IgG. Furthermore, we observed that the positivity rate for *B. burgdorferi* was significantly higher than for *Rickettsia* (Figure 9a). The positivity rate of *B. burgdorferi* was significantly higher in individuals under 20 years of age compared to those aged 21–40 and over 60, whereas no such difference was observed for *Rickettsia* (Figure 9c). No significant differences in IgG positivity rates for either pathogen were found between sex (Figure 9b).

## Discussion

### The impact of six factors on tick microbial communities

Previous studies have shown that microbial communities differ between free-living and parasitic ticks, with higher infection rates in parasitic stages (Swei and Kwan, 2017; Jiang et al., 2019). Our results confirm significant changes and a decline in microbial diversity from early parasitism to the over-engorged state, consistent with other studies comparing free-living and parasitic ticks (Brinkerhoff et al., 2020). Additionally, pathogen detection in the same batch of samples revealed that the infection rates of *Ehrlichia* and *Hepatozoon* increased significantly in over-engorged *R. sanguineus*, suggesting enhanced vectorial capacity due to blood-feeding (Eisen, 2018). However, for the other four pathogens, infection rates did not increase post-overfeeding, indicating that blood meal factors may have little impact on the transmission of these pathogens, or that their transmission times are too short, such as in the case of Powassan virus (Eisen, 2018). Our findings refine the understanding of microbial community changes from free-living to over-engorged states, as well as from early parasitism to over-engorged stages. Future research should explore these changes, quantify vectorial capacity at different feeding stages, and identify critical nodes in the blood meal process for intervention.

The study found significant differences in microbial communities between nymphs and adults of *R. sanguineus* and *H. hystricis*, with a decline in diversity, consistent with other studies (Zolnik et al., 2016; Chicana et al., 2019). As *R. sanguineus* develops, its microbial communities shift from “high individual diversity and uniform function” to “low individual diversity and diverse function,” a trend seen in other tick species, suggesting it may be a common survival strategy (Van Overbeek et al., 2008). Pathogen detection shows that different developmental stages affect vectorial capacity, with some pathogens more prevalent in adults (Reis et al., 2011) and others more common in nymphs, indicating stage-specific vectorial capacities (Clover and Lane, 1995).

Male *R. sanguineus* ticks have higher microbial diversity than females. Although this phenomenon was not observed in *H. hystricis*, other studies have demonstrated similar differences in other tick species (Van Treuren et al., 2015; Duncan et al., 2022). Female *R. sanguineus* ticks showed higher *Hepatozoon* infection rates, likely related to their longer parasitic periods. No significant sex differences were observed for other pathogens. Studies suggest that different pathogens may prefer specific sexes, thereby influencing the vectorial capacity of ticks (Kumar et al., 2007; Tappe et al., 2014; Kybicová et al., 2017). Additionally, sex may interact with other factors, affecting microbial communities and vectorial capacity (Batool et al., 2021).

Different tick species have varying vectorial capacities, with some species considered more medically significant (Jongejan and Uilenberg, 2004). This study observed differences at multiple levels between *R. sanguineus* and *H. hystricis,* particularly in *Ehrlichia* pathogen positivity rates, consistent with previous research (Brinkerhoff et al., 2020). Both species showed *Macrococcus*, *Staphylococcus*, and *Coxiella* as dominant genera under all conditions. In other regions, *R. sanguineus* microbial communities are dominated by *Coxiella*, *Rickettsia*, and *Bacillus* (Lalzar et al., 2012; René-Martellet et al., 2017), while in other countries, *Cetobacterium*, an unidentified genus under Corynebacteriaceae, and *Stenotrophomonas* dominate (Agwunobi et al., 2021). Prior research on Hainan Island found that *Staphylococcus* and *Coxiella* dominate *R. sanguineus* communities, confirming the local tick microbiome’s characteristics and differences from other regions (An et al., 2022). Our study, however, noted a significantly higher relative abundance of *Macrococcus* in Hainan (An et al., 2022). These findings suggest that the microbial community characteristics of the same tick species can vary across regions and that factors beyond geography influence these communities. Thus, assessing tick microbial communities and vectorial capacities under different conditions is essential for effective tick control strategies.

Existing studies have reported fluctuations in tick pathogen positivity rates and microbial communities over time (Lalzar et al., 2012; Mysterud et al., 2013; Beck et al., 2014). Our study observed variations in *Ehrlichia* and *Hepatozoon* positivity rates across time points in the SY and CZ regions, indicating temporal fluctuations in microbial communities and their potential association with vectorial capacity. Microbial diversity and function in June differed significantly from March and July. Similar fluctuations were observed in *Ixodes ricinus*, where microbial communities formed distinct clusters between March–April and May–September (Lejal et al., 2021). However, we found that *R. sanguineus* microbial community structure was similar in March and July but significantly different in June, likely due to tick species and geographical factors. These results suggest that tick microbial communities exhibit periodic fluctuations over time. Further studies are necessary to explore the causes and impacts of these fluctuations on vectorial capacity. Our findings support the link between seasonal microbial community changes and tick-borne pathogen transmission capacity, particularly in ticks from Hainan Island.

Some studies indicate that regional factors affect tick microbial communities (Kueneman et al., 2021), while geographical differences may influence tick vectorial capacity (Krawczyk et al., 2022). However, other research shows minimal (Brinkerhoff et al., 2020) or no impact (Chicana et al., 2019) of regional factors on tick microbial communities. These contradictions may arise from simplifying various factors like temperature, humidity, soil, vegetation, and hosts into “geographical factors” (Couret et al., 2022), complicating cross-study comparisons. Future research should further quantify “geographical factors,” as Van Treuren et al. explored the nonlinear relationship between physical distance and tick microbial communities by quantifying sampling site distances (Van Treuren et al., 2015).

### Overlooked transient species in microbial communities

Previous studies have typically considered tick microbial communities as a whole. Interestingly, by separating these communities into stable species and transient species, we observed that both types of species respond to six different factors at the species level. Their functional response patterns can be categorized into three modes: responses driven solely by stable species (during developmental stages), responses driven solely by transient species (during blood-feeding), and responses driven jointly by both (in response to sex, time, geography, and tick species). This indicates that various factors differentially affect specific components of tick microbial communities. Our study is the first to demonstrate that the selection and assembly processes of these microbial communities are not random but are influenced by different factors. Notably, transient species, previously thought to be environmental contaminants in ticks, play a significant role. Subsequent research suggests that ticks might actively incorporate these environmental microbes into their communities (Egyed and Makrai, 2014), and our study further confirms this phenomenon. The non-random nature of this assembly process and its directionality are crucial areas for future research. Currently, there is insufficient focus on these low-frequency and low-abundance transient species in the field of tick research, but our findings reveal their importance. Future studies should investigate the mechanisms by which ticks selectively incorporate environmental microbes into their communities and the direction of microbial community iteration.

### List of potential biomarkers

Using multiple differential analysis methods, we identified 73 robust differential species and 200 robust core species. After removing duplicates, 98 unique species were pinpointed as potential biomarkers for tick and tick-borne disease control. Some species serve as biomarkers in single groups, while others span multiple groups, suggesting broad functional spectra in ticks across various states. These species, though widely distributed in natural environments, have been largely overlooked by other studies. Notably, Zhong et al. (2021) found that *Coxiella*, a core species in *Haemaphysalis longicornis*, is key to blood-feeding behavior and blood volume regulation. In our study, *Coxiella* was also a core species in overfed *H. hystricis*, indicating similar functions, although it was absent in the blood-feeding stages of *R. sanguineus*, suggesting species-specific roles. *Coxiella* was identified across multiple dimensions, implying its role extends beyond blood-feeding. This study is the first to propose that *Coxiella* might influence other phenotypes. Despite its wide presence in hard ticks (Lalzar et al., 2012; Lalzar et al., 2014; Machado-Ferreira et al., 2016), *Coxiella*’s functions remain under-researched. *Staphylococcus* was also frequently identified and may serve as a versatile species with potential utility. Species found under multiple conditions could act as “broad-spectrum biomarkers” for tick and disease control, while those identified in specific states might be useful for targeted interventions.

### The epidemiological status of tick-borne pathogens in Hainan Island

This study detected six genera of tick-borne pathogens, which have been reported in Hainan Island over the past decades (Zheng et al., 2023). Our findings update these results, revealing a persistent risk of tick-borne diseases in the region. We analyzed the positive rates of pathogens in different groups, demonstrating their uneven distribution and preference for certain tick conditions. This suggests that six biotic and abiotic factors may influence tick vectorial capacity, potentially linked to microbial community changes. To explore the exposure level of tick-borne diseases in the Hainan population, we conducted IgG antibody tests for *Rickettsia* and *B. burgdorferi*. Results showed high exposure levels among the population. Previous studies have shown high exposure to *B. burgdorferi* in Hainan (Zhang et al., 2021). However, we did not detect *B. burgdorferi* in tick samples. As previous studies have shown that *B. burgdorferi* has a high positivity rate in small rodents (Žákovská et al., 2021), but there are also studies indicating higher infection rates in domestic animals such as dogs and cattle (He et al., 2022). Additionally, other research has detected *B. burgdorferi* in *R. sanguineus* and *H. hystricis*, suggesting that these tick species can serve as vectors for *B. burgdorferi* (Khoo et al., 2017; López-Pérez et al., 2019) Notably, a model by L. Zhang et al. predicted that the high-risk areas for Lyme disease in Hainan Island are mainly concentrated in the northern, central, and southern regions (Zhang et al., 2021). Although our study covered these sampling points, *B. burgdorferi* was still not detected in the tick samples. Therefore, we speculate that other reservoir hosts, vector species, or transmission pathways may be involved. This might be related to the limitations of our current samples. To address this issue, future studies will need to test a wider range of hosts and tick species to clarify these potential transmission pathways. A 2009–2010 study on 117 cases of unexplained fever in Hainan showed about 15% *Rickettsia* infection, indicating a high exposure risk and threat (Wu et al., 2014). These results suggest the persistent presence of *Rickettsia* and *B. burgdorferi* in Hainan’s environment. More extensive geographic distribution, multiple tick species, various hosts, and larger sample sizes are needed to map tick-borne pathogen circulation. The high exposure risk also calls for enhanced testing and proactive diagnosis of symptoms among residents.

## Conclusion

The results of this study illustrate the impact of various factors on tick microbial communities, describing the characteristics of microbial communities in different states and the non-random succession of transient species within these communities. The study also inferred the vectorial capacity of ticks in various states based on pathogen positivity rates, suggesting that these factors might influence the vectorial capacity of ticks. However, the vectorial capacity of ticks is influenced by various factors, with each factor affecting it differently. Additionally, the vectorial capacity of ticks varies for different pathogens. These six factors also impact tick microbial communities across different dimensions, including species composition, community function, and structure, necessitating more detailed research. Additionally, this study partly delineates the relatively high risk of tick-borne pathogens on Hainan Island from both the vector and human perspectives. Finally, a list of potential biomarkers was proposed, providing specific recommendations for developing strategies to control ticks and tick-borne diseases.

## Data Availability

The datasets presented in this study can be found in online repositories. The names of the repository/repositories and accession number(s) can be found in the article/Supplementary material.

## Supplementary material

Supplementary Figure S1 Species abundance and stability at phylum and genus levels in *R. sanguineus* and *H. hystricis*.

Supplementary Figure S2 Comparative analysis of species and functions via venn diagrams in *R. sanguineus* and *H. hystricis*.

Supplementary Figure S3 Robust differential species and functions in *R. sanguineus* and *H. hystricis*.

Supplementary Figure S4 Species level and functional level separate PCoA of transient and stable species.

Supplementary Figure S5 Robust core species across conditions in *R. sanguineus* and *H. hystricis*.

Supplementary Table S1 Sampling cites.

Supplementary Table S2 Group data and sequencing data table.

Supplementary Table S3 List of primers used for the detection of pathogens.

Supplementary Table S4 Differential analysis across various dimensions.

Supplementary Table S5 Potential biomarker list.

Supplementary Table S6 Detailed positive rates and differential analysis by group for tick-borne pathogens.

Supplementary File S1 Ticks COXI gene sequences.

Supplementary File S2 Stable and transient species.

Supplementary File S3 Robust differential species.

Supplementary File S4 Robust differential function.

Supplementary File S5 Node and edge information for microbial networks.

Supplementary File S6 Robust core species.
