# Supplementary figures and images for: Microbial community characteristics and pathogens detection in Rhipicephalus sanguineus and Haemaphysalis hystricis from Hainan Island, China
Source: Front Microbiol. 2024 Oct 8;15:1450219. doi: 10.3389/fmicb.2024.1450219 (PMC11493706; doi:10.3389/fmicb.2024.1450219)

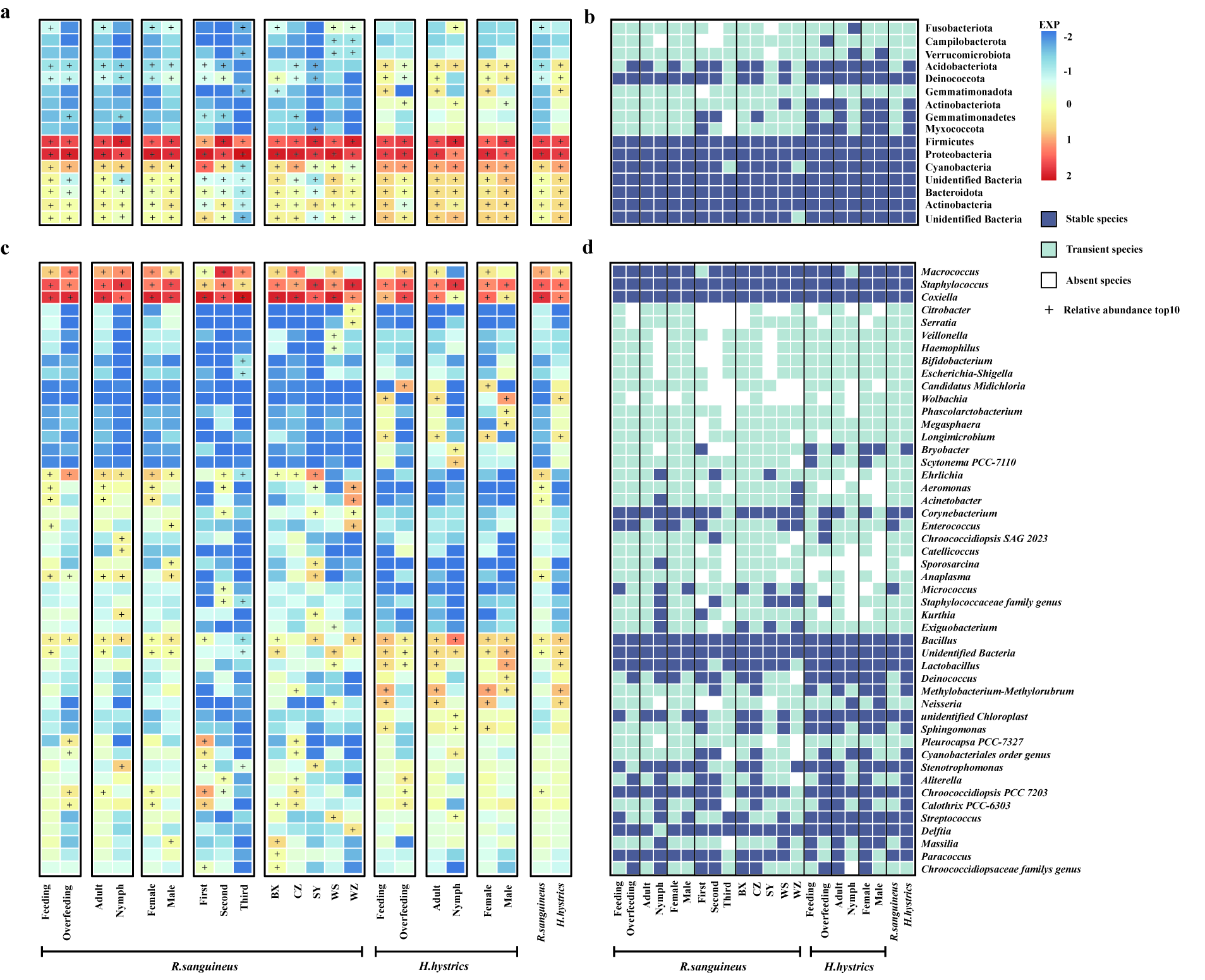

Supplement: Supplementary file 1 [file Data_Sheet_1.ZIP › Supplementary figure 1-5/Supplementary figure 1.tif]

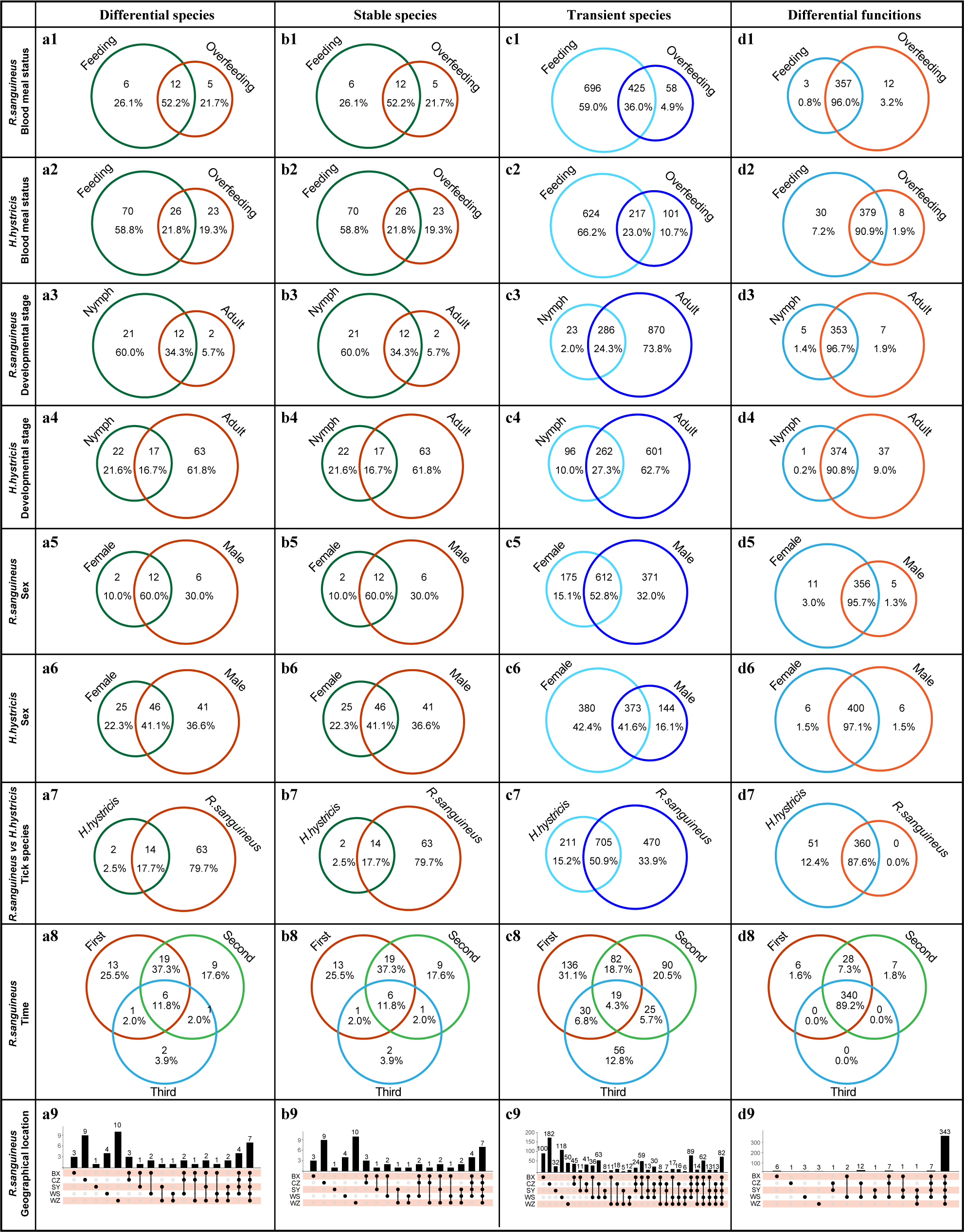

Supplement: Supplementary file 1 [file Data_Sheet_1.ZIP › Supplementary figure 1-5/Supplementary figure 2.tif]

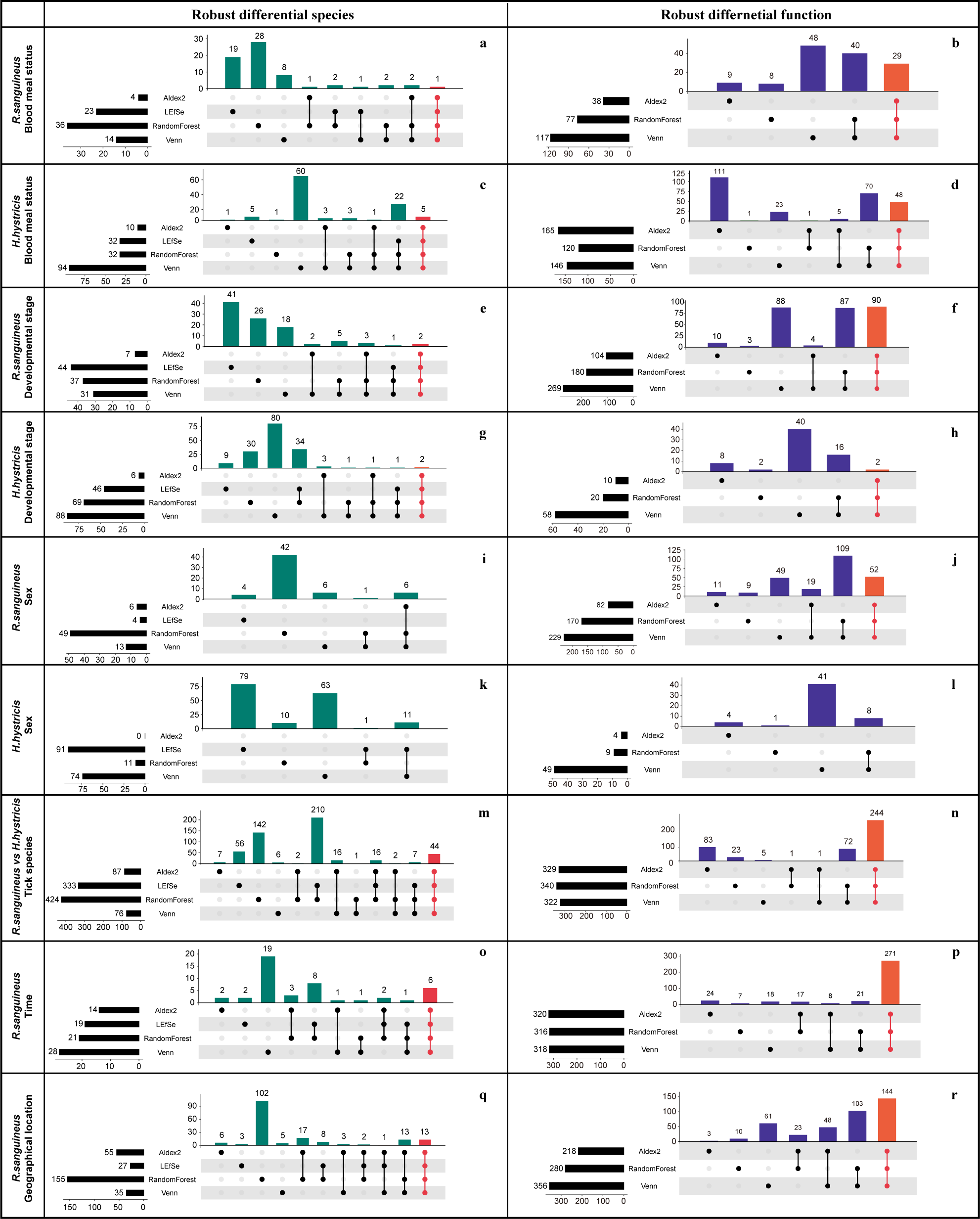

Supplement: Supplementary file 1 [file Data_Sheet_1.ZIP › Supplementary figure 1-5/Supplementary figure 3.tif]

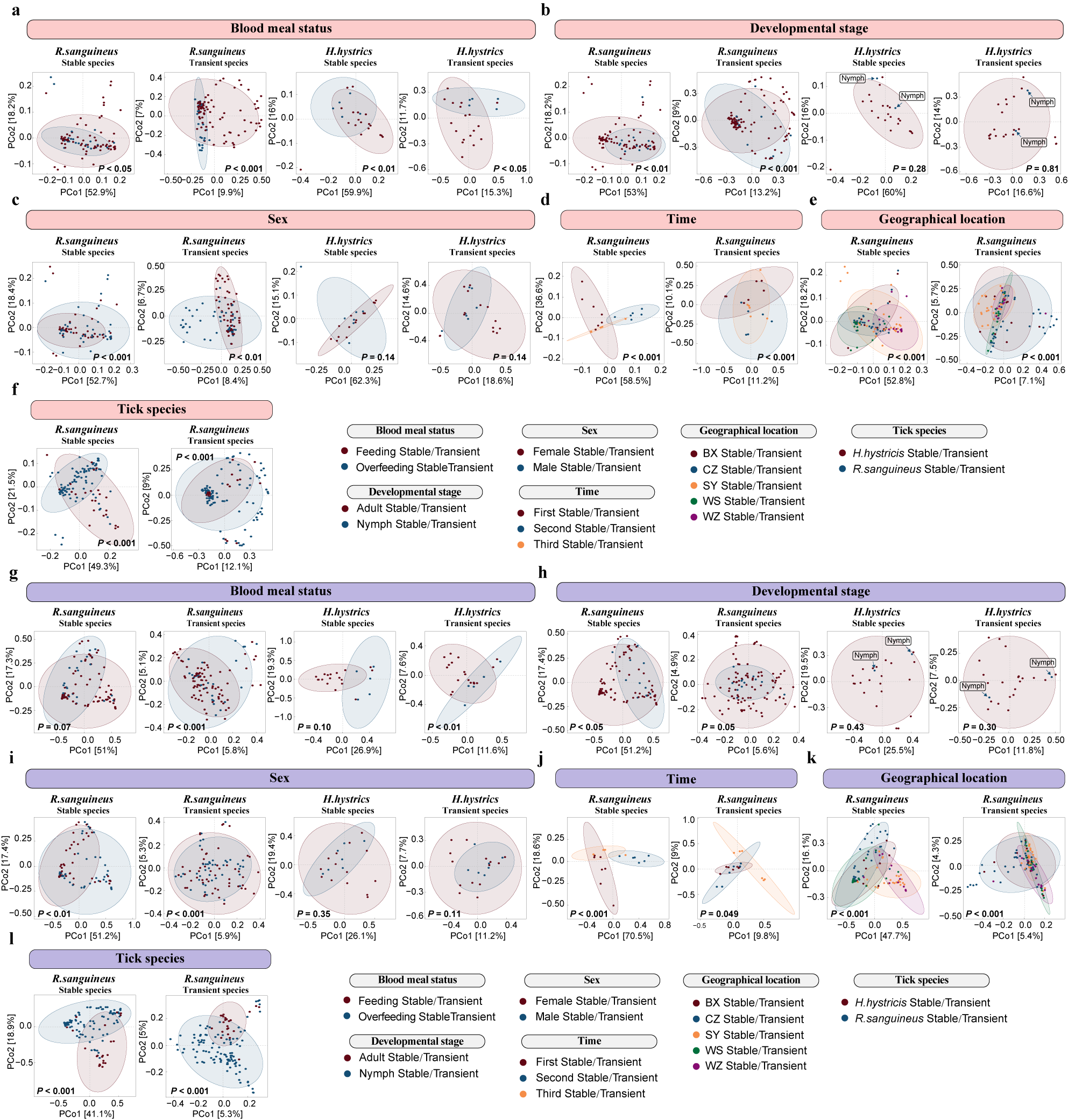

Supplement: Supplementary file 1 [file Data_Sheet_1.ZIP › Supplementary figure 1-5/Supplementary figure 4.tif]

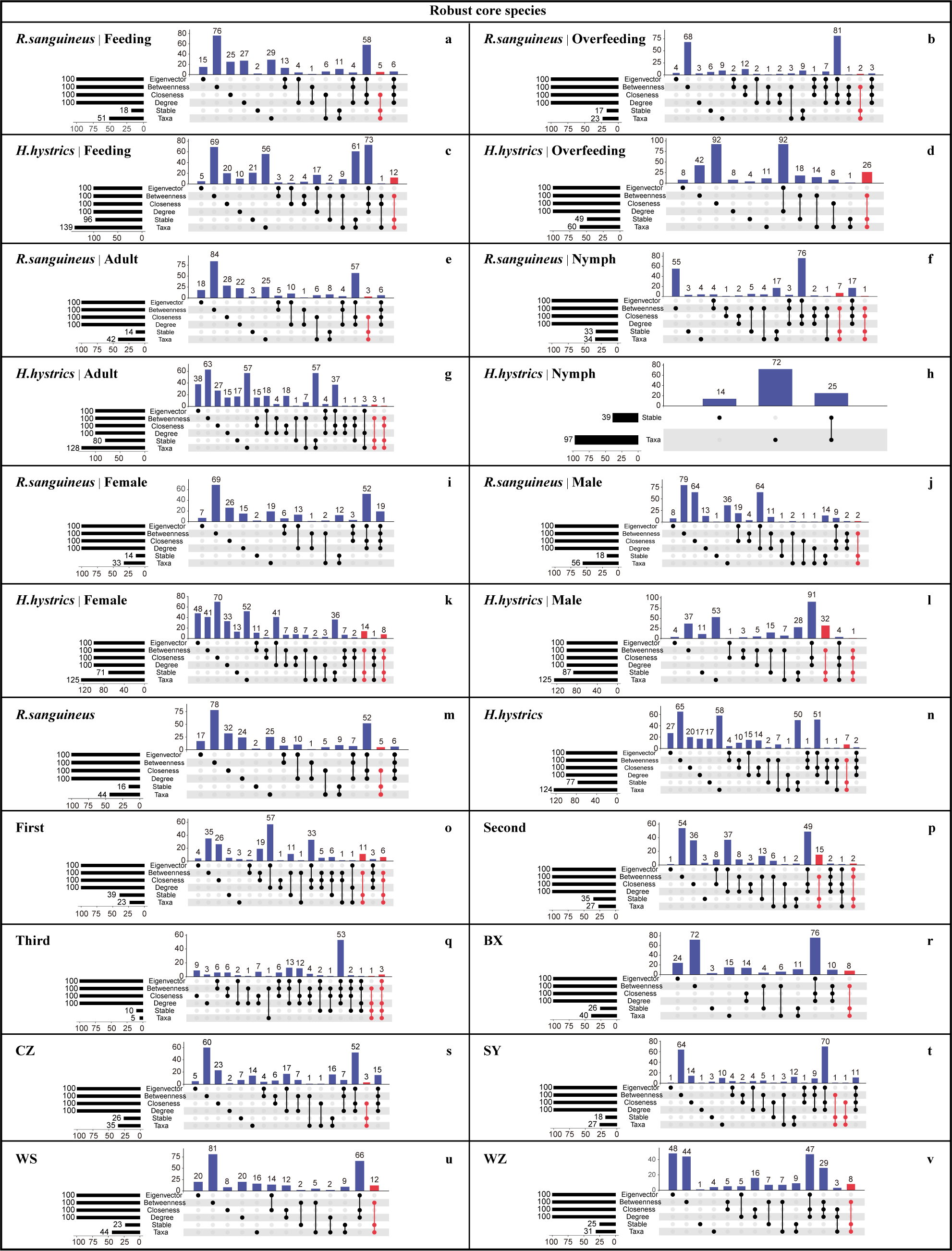

Supplement: Supplementary file 1 [file Data_Sheet_1.ZIP › Supplementary figure 1-5/Supplementary figure 5.tif]
