# Supplementary material for: Microbial community characteristics and pathogens detection in Rhipicephalus sanguineus and Haemaphysalis hystricis from Hainan Island, China
Source: Front Microbiol. 2024 Oct 8;15:1450219. doi: 10.3389/fmicb.2024.1450219 (PMC11493706; doi:10.3389/fmicb.2024.1450219)
Supplement: Supplementary file 3 [file Data_Sheet_3.ZIP › Supplementary table 1-6/Supplementary table 1_Sampling cites.docx]

**Supplementary Table 1：Sampling Points and Host Information**

| **Cites full name** | **Cite abbreviation** | **Coordinates** | **Host** |
| --- | --- | --- | --- |
| Lindan Village | LD | N 20° 3′ 15′′, E 110°23′16″ | Canine, Goat |
| Jiangyuan Village | JY | N 19° 58′ 40′′, E 110°17′23″ | Canine |
| Bangxi Town | BX | N 19°22′7″, E 109°6′10″ | Canine |
| Wanzhong Village | WS | N 19°6′31.18″, E 109°5′11″ | Canine |
| Changzheng Farm | CZ | N 18°56′39′′, E 109°55′1″ | Canine |
| Wanzhong Village | WZ | N 18°57′23″, E 109°50′19″ | Canine |
| Sanya City | SY | N 18°22′25.27″, E 109°42′31.23″ | Canine |
| Yinggen Town | YG | N 19°4′53.19″, E 109°46′5″ | Canine |
