# Supplementary material for: Microbial community characteristics and pathogens detection in Rhipicephalus sanguineus and Haemaphysalis hystricis from Hainan Island, China
Source: Front Microbiol. 2024 Oct 8;15:1450219. doi: 10.3389/fmicb.2024.1450219 (PMC11493706; doi:10.3389/fmicb.2024.1450219)
Supplement: Supplementary file 3 [file Data_Sheet_3.ZIP › Supplementary table 1-6/Supplementary table 3_List of primers used for the detection of pathogens.docx]

**Supplementary table 3. List of primers used for the detection of pathogens**

| **Pathogens** | **Gene** | **Primer** | **Sequences（5`→3`）** | **Tm（℃）** | **Length** | **Cite** |
| --- | --- | --- | --- | --- | --- | --- |
| *B. burgdorferi* | *Flab* | Borr-Bor1 | TAATACGTCAGCCATAAATGC | 49.7 | 753bp | [1] |
|  |  | Borr-Bor2 | GCTCTTTGATCAGTTATCATTC | 48.3 |  |  |
| *Rickettsia* spp. | *sca4* | Rick-D767f | CGATGGTAGCATTAAAAGCT | 49.7 | 626bp | [2] |
|  |  | Rick-D1390r | CTTGCTTTTCAGCAATATCAC | 49.2 |  |  |
| *Ehrlichia* spp.&*Anaplasma* spp | *16SrRNA* | Ehr/Ana-16S8FE | GGAATTCAGAGTTGGATCMTGGYTCAG | 59.4 | 500bp | [3] |
|  |  | Ehr/Ana-BGA1B | CGGGATCCCGAGTTTGCCGGGACTTCTTCT | 69.5 |  |  |
| *Babesia* spp.&*Theileria* spp. | *18SrRNA* | babe-RLB-F2 | GACACAGGGAGGTAGTGACAAG | 57.4 | 460-540bp | [4] |
|  |  | babe-RLB-R2 | CTAAGAATTTCACCTCTGACAGT | 51.5 |  |  |
| *Hepatozoon* spp. | *18SrRNA* | Hepa-HepF | ATACATGAGCAAAATCTCAAC | 47.3 | 660bp | [5] |
|  |  | Hepa-HepR | CTTATTATTCCATGCTGCAG | 48.1 |  |  |

1. Zhang, X.-A., et al., *Molecular detection and identification of relapsing fever Borrelia in ticks and wild small mammals in China.* Emerging Microbes & Infections, 2022. **11**(1): p. 2632-2635.

2. Levytska, V.A., et al., *Detection of pathogens in ixodid ticks collected from animals and vegetation in five regions of Ukraine.* Ticks and tick-borne diseases, 2021. **12**(1): p. 101586.

3. Chaisi, M.E., et al., *Comparison of three nucleic acid-based tests for detecting Anaplasma marginale and Anaplasma centrale in cattle.* Onderstepoort Journal of Veterinary Research, 2017. **84**(1): p. 1-9.

4. Norouzi, M., M.S. Dayer, and F. Ghaffarifar, *Molecular detection and characterisation of Theileria in hard ticks of small ruminants in Zarrin Dasht County, Southern Iran.* Veterinary Medicine and Science, 2023. **9**(1): p. 372-379.

5. Otranto, D., et al., *Diagnosis of Hepatozoon canis in young dogs by cytology and PCR.* Parasites & vectors, 2011. **4**(1): p. 1-6.
