# Supplementary material for: Microbial community characteristics and pathogens detection in Rhipicephalus sanguineus and Haemaphysalis hystricis from Hainan Island, China
Source: Front Microbiol. 2024 Oct 8;15:1450219. doi: 10.3389/fmicb.2024.1450219 (PMC11493706; doi:10.3389/fmicb.2024.1450219)
Supplement: Supplementary file 3 [file Data_Sheet_3.ZIP › Supplementary table 1-6/Supplementary table 6_Detailed positive rates and differential analysis by group for tick-borne pathogens.docx]

**Supplementary table 6: Detailed positive rates and differential analysis by group for tick-borne pathogens**

| **Group name** | **Total** | ***Rickettsia* n (%)** | ***P*** | ***Ehrlichia* n (%)** | ***P*** | ***Anaplasma* n (%)** | ***P*** | ***Borrelia* n (%)** | ***P*** | ***Babesia* n (%)** | ***P*** | ***Theileria* n (%)** | ***P*** | ***Hepatozoon* n (%)** | ***P*** |
| --- | --- | --- | --- | --- | --- | --- | --- | --- | --- | --- | --- | --- | --- | --- | --- |
| *R.sanguineus* |  |  |  |  |  |  |  |  |  |  |  |  |  |  |  |
| Feeding | 498 | 3 (0.60) | 0.567 | 26 (5.22) | **0.002** | 6 (1.20) | 1 | 0 (0) | - | 5 (1.00) | 0.263 | 1 (0.20) | 0.469 | 18 (3.61) | **<0.0001** |
| Overfeeding | 185 | 0 (0) |  | 23 (12.43) |  | 2 (1.08) |  | 0 (0) |  | 4 (2.16) |  | 1 (0.54) |  | 30 (16.22) |  |
|  |  |  |  |  |  |  |  |  |  |  |  |  |  |  |  |
| Female | 362 | 1 (0.28) | 0.566 | 32 (8.84) | 0.211 | 3 (0.83) | 0.275 | 0 (0) | - | 7 (1.93) | 0.494 | 1 (0.28) | 1 | 37 (10.22) | **<0.0001** |
| Male | 238 | 2 (0.84) |  | 14 (5.88) |  | 5 (2.10) |  | 0 (0) |  | 2 (0.84) |  | 0 (0) |  | 11 (4.62) |  |
|  |  |  |  |  |  |  |  |  |  |  |  |  |  |  |  |
| Adult | 600 | 3 (0.50) | 1 | 46 (7.67) | **<0.0001** | 8 (1.33) | 0.605 | 0 (0) | - | 9 (1.50) | 0.61 | 1 (0.17) | **0.003** | 48 (8.00) | **0.002** |
| Nymph | 83 | 0 (0) |  | 3 (3.61) |  | 0 (0) |  | 0 (0) |  | 0 (0) |  | 1 (1.20) |  | 0 (0) |  |
|  |  |  |  |  |  |  |  |  |  |  |  |  |  |  |  |
| SY-First | 77 | 0 (0) | - | 18 (23.38) | **0.006** | 2 (2.60) | 1 | 0 (0) | - | 0 (0) | 0.158 | 0 (0) | - | 3 (3.90) | **<0.0001** |
| SY-Second | 59 | 0 (0) |  | 0 (0%) |  | 0 (0) |  | 0 (0) |  | 0 (0) |  | 0 (0) |  | 0 (0) |  |
| SY-Third | 149 | 0 (0) |  | 26 (17.45) |  | 4 (2.68) |  | 0 (0) |  | 6 (4.03) |  | 0 (0) |  | 30 (20.13) |  |
|  |  |  |  |  |  |  |  |  |  |  |  |  |  |  |  |
| CZ-First | 46 | 0 (0) | - | 0 (0) | - | 0 (0) | 0.511 | 0 (0) |  | 0 (0) | 0.511 | 0 (0) | - | 6 (13.04) | **0.025** |
| CZ-Second | 53 | 0 (0) |  | 0 (0) |  | 2 (3.77) |  | 0 (0) |  | 2 (3.28) |  | 0 (0) |  | 2 (3.77) |  |
| CZ-Third | 30 | 0 (0) |  | 0 (0) |  | 4 (13.33) |  | 0 (0) |  | 0 (0) |  | 0 (0) |  | 7 (23.33) |  |
|  |  |  |  |  |  |  |  |  |  |  |  |  |  |  |  |
| BX | 36 | 0 (0) | **<0.001** | 0 (0) | **<0.0001** | 0 (0) | 0.501 | 0 (0) | - | 0 (0) | 0.837 | 0 (0) | **0.03** | 0 (0) | **<0.0001** |
| CZ | 129 | 0 (0) |  | 0 (0) |  | 2 (1.55) |  | 0 (0) |  | 2 (1.55) |  | 0 (0) |  | 14 (10.85) |  |
| JY | 22 | 2 (9.09) |  | 0 (0) |  | 0 (0) |  | 0 (0) |  | 0 (0) |  | 0 (0) |  | 0 (0) |  |
| LD | 61 | 1 (1.64) |  | 5 (8.20) |  | 0 (0) |  | 0 (0) |  | 0 (0) |  | 2 (3.28) |  | 0 (0) |  |
| SY | 255 | 0 (0) |  | 44 (17.25) |  | 6 (2.35) |  | 0 (0) |  | 6 (2.35) |  | 0 (0) |  | 33 (12.94) |  |
| WS | 32 | 0 (0) |  | 0 (0) |  | 0 (0) |  | 0 (0) |  | 0 (0) |  | 0 (0) |  | 0 (0) |  |
| WZ | 148 | 0 (0) |  | 0 (0) |  | 0 (0) |  | 0 (0) |  | 1 (0.68) |  | 0 (0) |  | 0 (0) |  |
|  |  |  |  |  |  |  |  |  |  |  |  |  |  |  |  |
| *H.hystricis* |  |  |  |  |  |  |  |  |  |  |  |  |  |  |  |
| Feeding | 69 | 1 (1.45) | 1 | 0 (0) | - | 0 (0) | - | 0 (0) | - | 0 (0) |  | 0 (0) |  | 1 (1.45) | 0.401 |
| Overfeeding | 20 | 0 (0) |  | 0 (0) |  | 0 (0) |  | 0 (0) |  | 0 (0) |  | 0 (0) |  | 1 (5.00) |  |
|  |  |  |  |  |  |  |  |  |  |  |  |  |  |  |  |
| Female | 26 | 1 (3.85) | 0.361 | 0 (0) |  | 0 (0) | - | 0 (0) | - | 0 (0) | - | 0 (0) | - | 0 (0) | - |
| Male | 46 | 0 (0) |  | 0 (0) |  | 0 (0) |  | 0 (0) |  | 0 (0) |  | 0 (0) |  | 0 (0) |  |
|  |  |  |  |  |  |  |  |  |  |  |  |  |  |  |  |
| Adult | 72 | 1 (1.39) | 1 | 0 (0) | - | 0 (0) | - | 0 (0) | - | 0 (0) | - | 0 (0) | - | 0 (0) | **<0.0001** |
| Nymph | 17 | 0 (0) |  | 0 (0) |  | 0 (0) |  | 0 (0) |  | 0 (0) |  | 0 (0) |  | 2 (11.76) |  |
|  |  |  |  |  |  |  |  |  |  |  |  |  |  |  |  |
| *R.sanguineus* | 683 | 3 (0.44) | 0.388 | 49 (7.17) | **0.004** | 8 (1.17) | 0.607 | 0 (0) | - | 9 (1.32) | 0.608 | 2 (0.29) | 1 | 48 (7.03) | 0.107 |
| *H.hystricis* | 89 | 1 (1.12) |  | 0 (0) |  | 0 (0) |  | 0 (0) |  | 0 (0) |  | 0 (0) |  | 2 (0) |  |

The Group name column includes comparisons across six dimensions. Three dimensions (blood meal status: feeding and overfeeding; gender: female and male; developmental stage: adult and nymph) are compared for both tick species (*R.sanguineus* and *H.hystricis*). Two dimensions (geographical location and time) are specific to *R.sanguineus*. The geographical locations include seven sites (BX, CZ, JY, LD, SY, WS, and WZ), with LD representing ticks parasitizing on goats and the other sites representing ticks parasitizing on dogs. The time dimension includes six time points for two locations: SY-First (2022-06-15), SY-Second (2022-07-08), SY-Third (2022-07-21) for SY; and CZ-First (2022-03-09), CZ-Second (2022-06-17), CZ-Third (2022-07-06) for CZ. The final dimension compares the positive rates between the two tick species. Statistical analyses were conducted using the Monte Carlo method for geographical location comparisons and Fisher's exact test for all other group comparisons.
